# Optogenetic control of Wnt signaling models cell-intrinsic embryogenic patterning using 2D human pluripotent stem cell culture

**DOI:** 10.1242/dev.201386

**Published:** 2023-07-26

**Authors:** Nicole A. Repina, Hunter J. Johnson, Xiaoping Bao, Joshua A. Zimmermann, David A. Joy, Shirley Z. Bi, Ravi S. Kane, David V. Schaffer

**Affiliations:** ^1^ Department of Bioengineering, University of California, Berkeley, CA 94720, USA; ^2^ Graduate Program in Bioengineering, University of California, San Francisco and University of California, Berkeley, CA 94720, USA; ^3^ Department of Chemical and Biomolecular Engineering, University of California, Berkeley, CA 94720, USA; ^4^ Gladstone Institute of Cardiovascular Disease, Gladstone Institutes, San Francisco, CA 94158, USA; ^5^ School of Chemical and Biomolecular Engineering, Georgia Institute of Technology, Atlanta, GA 30332, USA; ^6^ Helen Wills Neuroscience Institute, University of California, Berkeley, CA 94720, USA; ^7^ Department of Molecular and Cell Biology, University of California, Berkeley, CA 94720, USA

**Keywords:** Wnt signaling, Differentiation, Heterogeneity, Morphogenesis, Optogenetics, Patterning

## Abstract

In embryonic stem cell (ESC) models for early development, spatially and temporally varying patterns of signaling and cell types emerge spontaneously. However, mechanistic insight into this dynamic self-organization is limited by a lack of methods for spatiotemporal control of signaling, and the relevance of signal dynamics and cell-to-cell variability to pattern emergence remains unknown. Here, we combine optogenetic stimulation, imaging and transcriptomic approaches to study self-organization of human ESCs (hESC) in two-dimensional (2D) culture. Morphogen dynamics were controlled via optogenetic activation of canonical Wnt/β-catenin signaling (optoWnt), which drove broad transcriptional changes and mesendoderm differentiation at high efficiency (>99% cells). When activated within cell subpopulations, optoWnt induced cell self-organization into distinct epithelial and mesenchymal domains, mediated by changes in cell migration, an epithelial to mesenchymal-like transition and TGFβ signaling. Furthermore, we demonstrate that such optogenetic control of cell subpopulations can be used to uncover signaling feedback mechanisms between neighboring cell types. These findings reveal that cell-to-cell variability in Wnt signaling is sufficient to generate tissue-scale patterning and establish a hESC model system for investigating feedback mechanisms relevant to early human embryogenesis.

## Teaser

**Highlighted Article:** Optogenetic activation of Wnt signaling in human embryonic stem cells allows control of cell-to-cell variability, induces self-organization and tissue-scale patterning, and enables investigation of signaling feedback mechanisms.

## INTRODUCTION

Embryonic morphogenesis, a process where seemingly identical cells differentiate and organize into spatially defined regions, is regulated by dynamic, spatially and temporally varying patterns of signaling (Wennekamp et al., 2013). Cell-intrinsic differences in gene expression and cellular state (Eldar and Elowitz, 2010; Snijder and Pelkmans, 2011), as well as extrinsic asymmetries within the cell environment (Arnold and Robertson, 2009; Etoc et al., 2016), elicit signaling variability among individual cells, which subsequently changes their migration properties, cell-cell interactions and/or cell polarity to drive the coordinated organization of cells at a population level (Lecuit and Lenne, 2007; Steinberg and Takeichi, 1994).

Self-organization within the epiblast tissue of the mammalian embryo is first evident during gastrulation, where subpopulations of cells reorganize and differentiate along distinct cell lineages to form the three germ layers of the future organism (Arnold and Robertson, 2009). An asymmetric distribution of molecular signals (e.g. Wnt, Nodal, BMP and FGF) arises across the epiblast to activate posterior epiblast cells to undergo mesendoderm differentiation and an epithelial-to-mesenchymal transition (EMT) (Brennan et al., 2001; Ciruna and Rossant, 2001; Lawson et al., 1999; Liu et al., 1999), yielding a cell subpopulation that migrates away from the epithelial epiblast cells in the region of the primitive streak (Arnold and Robertson, 2009; Dumortier et al., 2012; Rivera-Pérez and Magnuson, 2005; Saykali et al., 2019).

The origin of this cell and tissue asymmetry, however, remains an open question. Studies of mouse embryogenesis and *in vitro* model systems show that Wnt signal induction from a localized source in extra-embryonic tissue breaks anterior-posterior symmetry of the epiblast (Harrison et al., 2017; Rivera-Pérez and Magnuson, 2005; Sozen et al., 2018). Specifically, the combination of BMP4 secreted from the posterior extra-embryonic ectoderm (Winnier et al., 1995) and secreted inhibitors from the anterior (Takaoka et al., 2011) induces localized expression of Wnt3 in the posterior epiblast, establishes a Nodal and Wnt (Nodal/Wnt) feedback loop, and induces mesendodermal differentiation and EMT in a fraction of epiblast cells (Ben-Haim et al., 2006; Rivera-Pérez and Magnuson, 2005; Sozen et al., 2018). The exact extra-embryonic source for Wnt induction, though, remains ambiguous (Ben-Haim et al., 2006; Sozen et al., 2021; Tortelote et al., 2013; Winnier et al., 1995; Yoon et al., 2015). Spatially asymmetric patterns of molecular signals and cell fate can also arise in the absence of extra-embryonic tissue entirely (Etoc et al., 2016; Simunovic et al., 2019; ten Berge et al., 2008; Turner et al., 2017; van den Brink et al., 2014; Vianello and Lutolf, 2021 preprint), implying that asymmetry could originate from other extrinsic sources such as mechanical cues (Blin et al., 2018; Muncie et al., 2020; Sagy et al., 2019). Alternatively, cell-intrinsic variability within a cell population can serve as a mechanism to differentially sensitize cells to a uniform extrinsic signal, and cells that accordingly adopt different fates can spatially self-segregate (Dietrich and Hiiragi, 2007; Mohammed et al., 2017; Ohnishi et al., 2014; Snijder et al., 2009; Suppinger et al., 2023). The relative relevance of these potential avenues for emergence of asymmetry in the mammalian embryo is not well understood.

Embryonic stem cell (ESC) culture systems that model mammalian, and particularly human, development have offered mechanistic insight into morphogenesis (Shahbazi and Zernicka-Goetz, 2018). Such *in vitro* models can show remarkable morphological and molecular similarity to natural embryos (Beccari et al., 2018; Rivron et al., 2018; Sozen et al., 2018), while enabling molecular perturbation to study mechanism. As one example, mouse ESCs (mESCs), originally derived from the pre-implantation blastocyst (Evans and Kaufman, 1981; Martin, 1981) and grown as aggregates called gastruloids, spontaneously form spatial patterns of signaling and initiate mesendoderm differentiation, an effect enhanced by an exogenous and spatially uniform pulse of Wnt agonist (ten Berge et al., 2008; Turner et al., 2017; van den Brink et al., 2014). Gastrulation-like events are also observed in mESC aggregates grown adjacent to extra-embryonic tissue, an effect suppressed by the Wnt antagonist Dkk1 (Girgin et al., 2021; Harrison et al., 2017; Sozen et al., 2018; Zhang et al., 2019). Excitingly, human ESCs (hESCs) have extended such developmental models from murine to human embryogenesis, which, due to ethical restrictions, has long been a mystery (Shahbazi and Zernicka-Goetz, 2018; Thomson et al., 1998). For example, hESCs geometrically confined to two-dimensional circular micropatterns self-organize into radially symmetric patterns of germ lineages in response to uniform addition of BMP4 or Wnt agonists (Etoc et al., 2016; Martyn et al., 2019; Warmflash et al., 2014), and establish signal feedback loops between Wnt, Nodal and BMP4 pathways (Martyn et al., 2018). More recently, 3D hESC gastruloid models have been reported, wherein a 2D population of hESCs is primed with a pulse of BMP or Wnt activation before being aggregated in 3D, and cells of the three germ layers emerge in the extending structures with striking similarity to the human gastrula (Moris et al., 2020; Simunovic et al., 2019; Zheng et al., 2019).

Exogenous molecular perturbation of ESC-based models and natural embryos, however, does not enable differential control of specific cell subpopulations. For example, signal agonists or inhibitors are typically applied uniformly and on a population level, making it difficult to determine the role of signaling within individual cells or cell subpopulations in the process of pattern formation. In addition, cell responses to uniform signaling inputs are confounded by variations in molecular diffusion of agonists within larger cellular structures (Carpenedo et al., 2009) and geometric asymmetries in the cell environment or culture system (Etoc et al., 2016; Blin et al., 2018; Harrison et al., 2017). Although such mechanisms for generating spatial asymmetry may also be relevant to *in vivo* morphogenesis, tools for more precise control of intrinsic cell-to-cell signaling variability would be beneficial but are lacking. It thus remains challenging to study how specific molecular signals direct the organization, fate specification and migration of specific cell subpopulations.

To address this crucial need for targeted control of morphogen signaling within specific subpopulations of cells, and to investigate the role of selective Wnt activation during early embryonic differentiation, we have developed an optogenetic approach to perturb Wnt signaling in hESCs. Light-sensitive protein domains that induce protein-protein interactions and/or modify protein activity in response to illumination allow optogenetic control of signaling with spatial and temporal precision (Deisseroth and Hegemann, 2017; Repina et al., 2017). Optogenetic strategies have been applied for spatiotemporal control of transcription and intracellular signaling in the *Drosophila* (Huang et al., 2017; Izquierdo et al., 2018; Johnson et al., 2017; Johnson and Toettcher, 2019; Singh et al., 2021) and zebrafish embryo (Čapek et al., 2019; Sako et al., 2016; Wang et al., 2010). Here, we harness our previously developed optogenetic Wnt signaling system (optoWnt) (Bugaj et al., 2013) in hESCs to determine whether cell-to-cell variability in Wnt signaling can model early stages of human gastrulation and lead to emergence of cellular organization. In particular, using wild-type (WT) hESCs and/or cells expressing the optoWnt system, a fusion of the plant blue-light photoreceptor Cryptochrome 2 (Cry2) to the Wnt co-receptor LRP6 (Bugaj et al., 2013), we found that Wnt activation within cell subpopulations drives cell differentiation and segregation in 2D cultures, a process dependent on apparent EMT and downstream activation of TGFβ signaling. Furthermore, transcriptomic analysis identified a BMP and TGFβ (BMP/TGFβ) signaling feedback between the mesenchymal and epithelial subpopulations. Overall, starting from a salt-and-pepper distribution, our hESC co-cultures are able to self-organize, suggesting that, even in the absence of spatially patterned cues, heterogeneous activation of Wnt is sufficient for self-organization and mesendoderm migration away from an epithelial cell population. The result is a human developmental model for studying Wnt-mediated EMT, morphogenesis and signaling feedback among cell subpopulations.

## RESULTS

### Optogenetic activation of Wnt/β-catenin signaling and differentiation in hESCs

Canonical Wnt/β-catenin signaling initiates when extracellular Wnt protein binding to the transmembrane receptor Frizzled triggers multimeric clustering of the Wnt co-receptor low-density lipoprotein receptor-related protein 6 (LRP6) (Bilić et al., 2007). LRP6 oligomers are subsequently phosphorylated and induce an intracellular signaling cascade that stabilizes the downstream Wnt effector β-catenin, which in turn transcriptionally activates target genes.

**Fig. 1.**
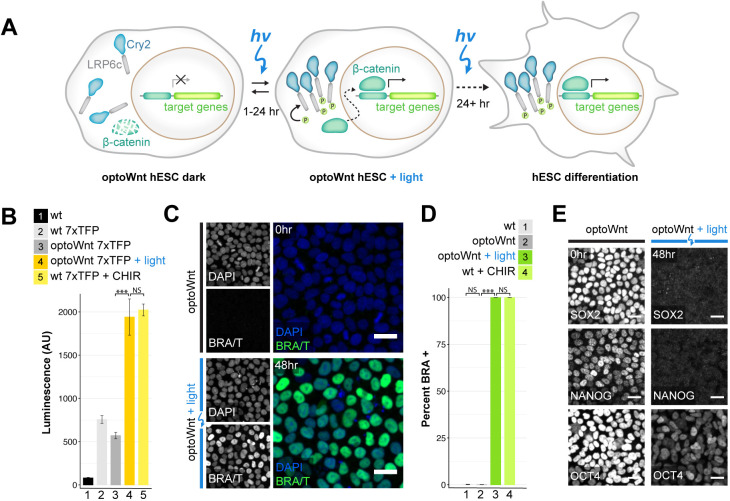
**Optogenetic activation of Wnt/β-catenin signaling and differentiation in hESCs.** (A) Schematic of optogenetic Wnt/β-catenin pathway activation (optoWnt) in hESCs. (B) Luciferase assay in WT and optoWnt hESCs carrying a 7xTFP reporter for β-catenin activity. Wnt signaling was induced for 24 h with 0.8 µW mm^−2^ illumination or with CHIR99021 (CHIR, 3 µM). ANOVA followed by Tukey's test [***P=4.6×10^−7^ (3 versus 4); *P*=0.86 (4 versus 5)]. Data are mean±s.d., *n*=3 biological replicates. (C) Representative images of immunostaining for brachyury in optoWnt hESC in the dark (top) or after 48 h illumination (bottom). Scale bars: 25 µm. (D) Quantification of immunostaining for brachyury in WT and optoWnt hESCs after 48 h illumination or CHIR (5 µM) treatment. Optogenetic stimulation induced a >99.9% pure population of BRA^+^ hESCs. ANOVA followed by Tukey's test [***P<1×10^−14^ (2 versus 3); *P*=0.28 (1 versus 2); *P*=0.97 (3 versus 4)]. Data are mean±s.d., *n*=3 biological replicates. (E) Representative images of immunostaining for pluripotency markers SOX2, NANOG and OCT4 in optoWnt hESCs kept in the dark (left) and after 48 h illumination (right). Scale bars: 25 µm.

We previously developed an optogenetic system consisting of the photolyase homology domain of the *A. thaliana* blue-light photoreceptor Cryptochrome 2 (Cry2) fused to the cytoplasmic domain of LRP6 (LRP6c) (Bugaj et al., 2013) (Fig. 1A), named ‘optoWnt’, which we subsequently knocked into the *AAVS1* safe harbor locus (Smith et al., 2008) using CRISPR/Cas9-mediated homology-directed repair to generate clonal hESC and induced pluripotent stem cell (iPSC) lines (Repina et al., 2020b). These clonal lines expressed Cry2-LRP6c for light-induced Wnt activation, retained a pluripotent phenotype, and showed uniform expression of P2A-linked mCherry for cell identification (Fig. S1A-F). Blue (470 nm) light stimulation of cell cultures was achieved with specialized LED illumination devices (light activation at variable amplitude, or LAVA, devices) (Repina et al., 2020a,b) that allow precise control of the intensity, timing and uniformity of stimulation (Fig. S1G-I).

Consistent with our previous work (Repina et al., 2020b), illumination of optoWnt hESCs induced LRP6 oligomerization due to clustering of activated Cry2, subsequent β-catenin protein localization to the nucleus (which is indicative of canonical Wnt signaling; Clevers et al., 2014) and activation of the β-catenin-responsive luciferase reporter 7xTFP (Fuerer and Nusse, 2010) (Fig. 1B, Fig. S1J-L). OptoWnt stimulation subsequently induced expression of the mesendoderm transcriptional regulator and primitive streak marker brachyury (BRA, also known by its gene symbol, *T*), a direct transcriptional target of Wnt signaling (Arnold et al., 2000; Rivera-Pérez and Magnuson, 2005; Yamaguchi et al., 1999), resulting in a greater than 99.9% pure BRA^+^ population with a marked decrease in expression of ESC pluripotency markers SOX2, NANOG and OCT4, which is indicative of cell differentiation (Davidson et al., 2012; Lindsley et al., 2006; Sumi et al., 2008) (Fig. 1C-E, Figs S1M-O, S2). Finally, optoWnt hESCs showed no detectable dark-state activation, as shown by a lack of LRP6 clusters, undetectable nuclear-localized β-catenin or BRA, low β-catenin transcriptional activity, and no changes in pluripotency marker expression (Fig. 1B-E, Figs S1J-O, S2).

### Global transcriptional profiling of optogenetic stimulation confirms light-induced mesendoderm lineage commitment

**Fig. 2.**
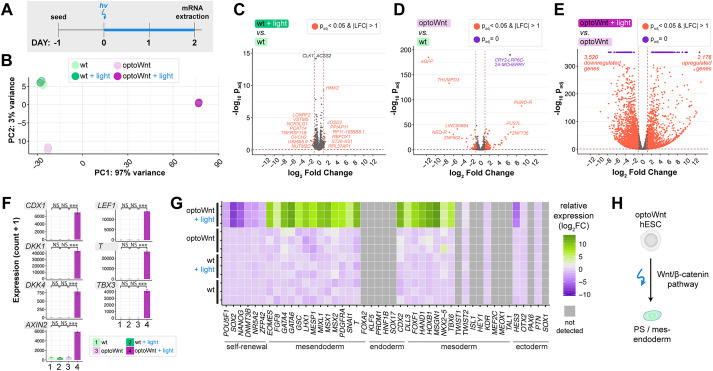
**RNA-seq of optoWnt hESCs shows light-induced mesendoderm differentiation, low phototoxicity and low optoWnt dark-state activity.** (A) Schematic of experimental timeline. Wild-type and optoWnt cells were kept in the dark or illuminated at 0.8 µWmm^−2^ for 48 h. (B) Principal component analysis (PCA) of RNA-seq results. Colors indicate the four experimental conditions. Each point is a biological replicate. (C-E) Volcano plots of RNA-seq differential expression analysis, with DEGs (adjusted *P*-value <0.05 and log_2_ fold change >1) in red and DEGs saturated at *P*=0 in purple. (C) Illuminated WT versus unilluminated WT hESCs (phototoxicity control); (D) unilluminated optoWnt versus unilluminated WT hESCs (dark-state activity control); (E) illuminated optoWnt versus unilluminated optoWnt hESCs. (F) β-Catenin target gene expression. ANOVA followed by Tukey's test. Graphs show mean expression (read counts+1)±s.d., *n*=3 biological replicates. (G) Heat map of log_2_ fold change in lineage markers normalized to unilluminated WT hESC expression level. Biological replicates displayed for each condition, with undetected genes (read count<150) shown in gray. (H) Schematic of light-induced mesendoderm differentiation.

To establish a molecular fingerprint for optoWnt-induced differentiation, we measured global transcriptional changes using bulk-population RNA-seq of optoWnt and WT hESCs after 48 h of light stimulation (Fig. 2A; Table S1). Illuminated WT cells served as a phototoxicity control, and unilluminated optoWnt cells controlled for potential CRISPR/Cas9 knock-in effects, cell perturbation due to optoWnt expression or dark-state Wnt pathway activation. Principal component analysis (PCA) showed clustering of biological triplicates for each condition and strong transcriptional changes upon optoWnt stimulation that account for 97% gene variance among samples (Fig. 2B). Dark and illuminated WT cells clustered together, and differential analysis showed minimal gene expression differences, demonstrating minimal phototoxicity effects after 48 h of continuous 0.8 µW mm^−2^ blue light stimulation (Fig. 2B,C). The remaining 3% of variance was captured by the second principal component, which accounted for slight transcriptional differences between the unilluminated WT versus optoWnt cells (Fig. 2D). Notably, none of the differentially expressed genes (DEGs, highlighted red) are members of the Wnt/β-catenin pathway, including downstream transcriptional target *AXIN2*, suggesting that transcriptional differences are due to gene knock-in or protein overexpression effects and not to background Wnt pathway activation.

In contrast, optoWnt stimulation had a broad transcriptional impact, with ∼5500 differentially expressed genes between the dark and illuminated conditions (Fig. 2E). Direct β-catenin target genes, such as *CDX1*, *DKK1*, *T* and *TBX3*, were among the most differentially expressed genes, all with a log fold change (LFC) of ∼9-13 (Fig. 2F, Fig. S3A,B). To determine whether optoWnt stimulation induced hESC differentiation along a mesendoderm lineage, we analyzed transcriptional changes in fate markers associated with embryonic germ layer specification (Fig. 2G, Fig. S3C,D). Illumination for 48 h induced strong upregulation of the primitive streak and mesendoderm markers *T*, *EOMES*, *MIXL1*, *GATA6*, *MSX1* and *GATA4* with a corresponding decrease in self-renewal markers *POU5F1*, *SOX2* and *NANOG*. Endodermal markers, such as *FOXA2* and *SOX17*, as well as ectodermal markers, such as *SOX1* and *PAX6*, remained low or were downregulated. Conversely, certain mesodermal markers, such as *TBX6*, *FOXF1* and *HOXB1*, were upregulated, consistent with a role for Wnt in inducing mesoderm differentiation in the absence of high TGFβ signaling (Martyn et al., 2018). In summary, bulk RNA-sequencing analysis confirmed that optogenetic stimulation of optoWnt hESCs induced robust mesendoderm differentiation with undetectable phototoxicity, low background activity and large dynamic range (Fig. 2H).

### Wnt signaling in spatially mixed cell subpopulations is sufficient to induce cell segregation in hESC culture

**Fig. 3.**
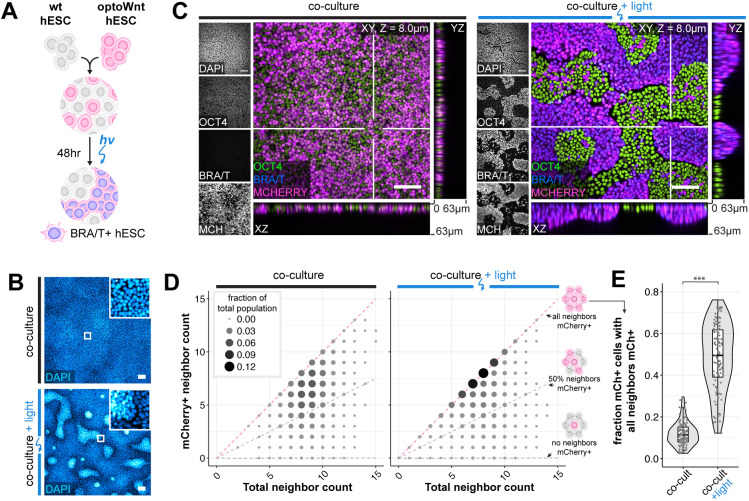
**Cell self-organization upon optoWnt stimulation of cell subpopulations.** (A) Schematic of experimental setup of optoWnt/WT hESC co-cultures. (B) Representative images of fixed optoWnt/WT co-cultures in the dark (top) and after 48 h of illumination (bottom) stained with DAPI. Scale bars: 100 µm. (C) Confocal images of optoWnt/WT co-cultures in the dark (left) and after 48 h illumination (right), stained for OCT4 and brachyury. OptoWnt cells are labelled with mCherry (mCh) expression. Scale bars: 100 µm. *yz* and *xz* axial cross-sections shown through indicated slices (white lines), 63 µm in height. (D) Cell neighbor analysis of optoWnt (mCh^+^) cells in co-cultures kept in the dark (left) or illuminated for 48 h (right). Graph shows the count of total cell neighbors versus the count of mCh^+^ cell neighbors across the total population of analyzed mCh^+^ cells (95,685 cells analyzed, pooled analysis from *n*=3 biological replicates). Area and color of points is proportional to the fraction of total population. Constant ratios of mCh^+^ to total neighbors are highlighted with pink and gray lines. (E) Quantification of the fraction of optoWnt (mCh^+^) cells whose neighbors are all mCh^+^. Each point represents an analyzed field of view (108 fields of view analyzed, *n*=3 biological replicates). Unpaired two-sample Wilcoxon test (***P<2.2×10^−16^). Box plots extend from 25th to 75th percentile; horizontal lines represent median; whiskers represent 1.5× interquartile range. The width of the shaded area (violin plot) represents the data density.

Equipped with a method for controlling mesendoderm differentiation, we investigated whether cell-to-cell variability in Wnt signaling could contribute to cell self-organization. Specifically, optoWnt and wild-type hESCs were mixed into a 1:1 heterogeneous culture such that illumination would activate Wnt signaling in only the optoWnt subpopulation of a hESC colony (Fig. 3A). When kept in the dark, the mCherry-positive optoWnt hESCs remained heterogeneously mixed with WT hESCs. Strikingly, illuminated co-cultures showed a strong segregation between the two cell populations, where optoWnt and WT cells separated and created sharp boundaries between the two resulting domains (Fig. 3B,C, Fig. S4A,B). This was a surprising observation as spontaneous Wnt activation has been reported in mESC gastrulation models but did not appear to cause a distinct spatial segregation between cell populations (Beccari et al., 2018; Harrison et al., 2017). In addition to segregating, axial cross-sections showed that WT cells remained in an epithelial monolayer, whereas optoWnt cells piled into vertical stacks up to ∼60 µm in height, apparently minimized contact area with WT cells by forming steep and spatially separated boundaries at domain edges, and displayed a mesenchymal morphology with increased cell protrusions and scattering of single cells from hESC colonies (Fig. 3C, Fig. S4C,D). In contrast, in illuminated monocultures, optoWnt cells piled vertically only in certain regions and with less defined boundaries (Fig. S4E). We tested different optoWnt:WT seeding ratios to determine whether an optoWnt dosage threshold existed for segregation, but we observed clear segregation at all ratios (Fig. S5A).

We next quantified the extent of segregation by counting the cell neighbors of optoWnt (mCherry+) cells (Fig. 3D, Fig. S4F,G). If the two populations are well mixed in a 1:1 ratio, a given optoWnt cell would be in contact with equal numbers of optoWnt versus WT neighbors, such that few would be surrounded by 100% optoWnt neighbors. In contrast, if the two populations are perfectly segregated, a substantially higher fraction of optoWnt cells (all cells away from population boundaries) would be fully surrounded by optoWnt neighbors. Indeed, dark co-cultures remained well mixed with a wide distribution of neighbor counts that were slightly skewed toward higher ‘like’ neighbor percentages, reflecting the clonal expansion that occurs over the 3-day experiment duration (Fig. 3D). In contrast, illuminated co-cultures displayed an approximately fourfold higher fraction of optoWnt cells that were entirely surrounded by cells of their own kind, a result consistent with cell segregation (Fig. 3D,E). Strong segregation was observed in all culture media conditions tested, including basal media lacking the pluripotency maintenance factors FGF2 and TGFβ, suggesting that Wnt signaling alone is sufficient to induce the observed cell self-organization (Fig. S6). These results suggest that cell-to-cell variability in Wnt signal activation is sufficient to generate tissue-scale patterning.

### Self-organization occurs through optogenetic induction of an apparently epithelial-to-mesenchymal transition and cell migration

**Fig. 4.**
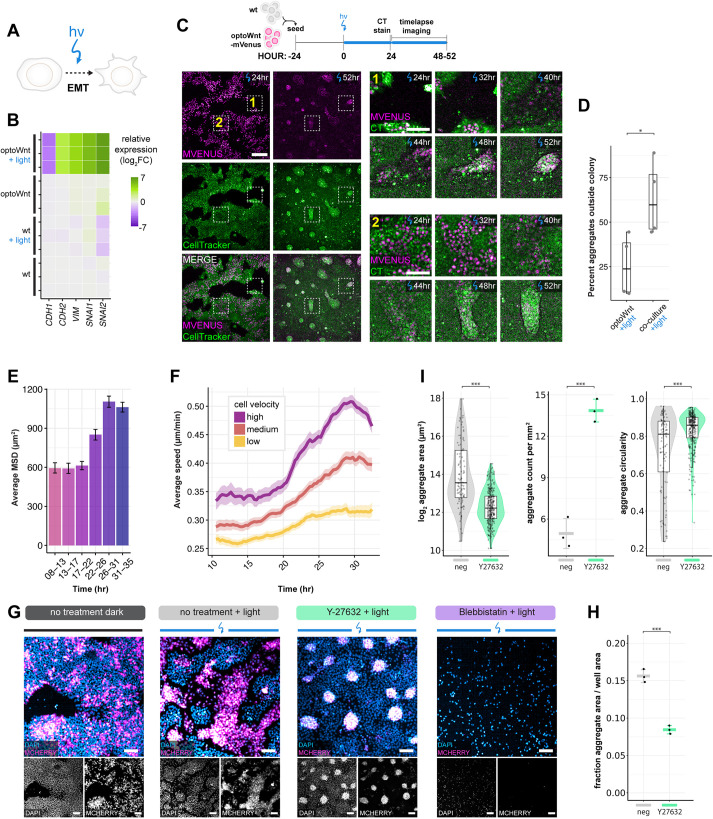
**Self-organization is mediated by Wnt-induced cell migration and EMT.** (A) Schematic of optogenetic induction of EMT. (B) RNA-seq heat map of log_2_ fold change in EMT marker genes normalized to mean gene expression of WT hESCs. Biological replicates are displayed for each condition. (C) Live-cell timelapse fluorescence imaging of optoWnt/WT co-cultures during optogenetic stimulation at the indicated time points. OptoWnt cells are labelled with mVenus-NLS expression, and all cells are labelled with CellTracker (CT) dye. Zoom in (right) of regions 1 (cell aggregation outside hESC colony) and 2 (cell aggregation inside hESC colony) at indicated time points. Scale bar: 250 µm (left); 100 µm (zoom in). (D) Percentage of aggregates forming outside hESC colonies (as shown in region 1) over total formed aggregates. Graph shows analysis of *n*=4 fields of view, one-way ANOVA (*P=0.034). (E) Average mean squared displacement (MSD) of single-cell trajectories of optoWnt cells in optoWnt/WT co-cultures at indicated time intervals after onset of light stimulation. Graph shows mean (>1000 tracks over 5 fields of view)±95% confidence interval. (F) Average cell speed over time of optoWnt single-cell trajectories binned by median cell velocity (low: 0-0.15 µm/min; medium: 0.15-0.18 µm/min; high: 0.18-0.6 µm/min). Data are mean±95% confidence interval. (G) Representative images of immunostaining for mCherry (optoWnt) and DAPI in optoWnt/WT co-cultures under indicated inhibitor conditions after 48 h illumination. Scale bars: 100 µm. (H) Quantification of optoWnt aggregate area normalized to imaged area after 48 h illumination under no treatment or with Y-27632 treatment. Graph shows analysis of *n*=3 wells, mean±s.d, one-way ANOVA (***P=0.000272). (I) Quantification of optoWnt aggregate morphology with aggregate area (log_2_ transformed, left, ***P<2×10^−16^), number of aggregates per unit area (middle, data are mean±s.d., ***P=0.0003), and aggregate circularity (right, ***P=5.5×10^−5^). Left and right graphs use the unpaired two-sample Wilcoxon test; middle graph uses one-way ANOVA. Box plots extend from 25th to 75th percentile; horizontal lines represent median; whiskers represent 1.5× interquartile range. The width of the shaded area (violin plot) represents the data density.

Cells of the primitive streak undergo an EMT (Dumortier et al., 2012; Scarpa and Mayor, 2016), a process regulated at least in part by Wnt signaling as mouse embryos mutant for *Wnt3* or *Ctnnb1* (β*-*catenin) do not ingress or form a primitive streak (Huelsken et al., 2000; Liu et al., 1999). To determine whether optoWnt activation is sufficient for EMT induction, we first assayed for EMT hallmarks upon light stimulation using bulk RNA-sequencing (Fig. 4A,B). E-cadherin (*CDH1*) levels markedly decreased after 48 h of illumination, while N-cadherin (*CDH2*) levels increased – a switch in cell-adhesion proteins that is characteristic of EMT (Scarpa and Mayor, 2016). In addition, other indicators of EMT, such as the cytoskeletal protein vimentin (*VIM*) and E-cadherin transcriptional repressors snail and slug (*SNAI1* and *SNAI2*) were upregulated. Similar trends were observed at the transcriptional level through qPCR and at the protein level with immunostaining for E- and N-cadherin (Fig. S7A-C). These results suggest that an EMT-like transition occurred in response to optoWnt activation.

EMT is often accompanied by a migratory cell phenotype (McDole et al., 2018; Saykali et al., 2019; Scarpa and Mayor, 2016) (Fig. S4C,D). As clear segregation of cell populations was observed upon optoWnt activation of hESC co-cultures, we analyzed the specific role that cell motility may play during the apparent EMT occurring in this self-organization process. Live cell timelapse imaging during light stimulation showed that optoWnt cells became increasingly migratory and led to visible aggregation after 30-40 h of illumination (Fig. 4C; Movie 1). We observed that segregation resulted from both (1) optoWnt^+^ cell migration out of the epithelial hESC colonies and aggregation in the empty space between colonies, and (2) optoWnt^+^ migration and aggregation within the colony (Fig. 4D; Movies 1 and 2). Once formed, the dynamic behavior of aggregates was particularly striking, with aggregates migrating rapidly as a group, merging with nearby aggregates or detaching from the plate surface entirely (Movie 3). We considered whether apparent segregation could be mediated by an increased proliferation of rapidly expanding subpopulations of optoWnt cells, but we observed no evidence of clonal expansion via live-cell imaging (Fig. S7D), and illumination of optoWnt cells in monoculture showed only a mild increase in proliferation rate (Fig. S7E,F).

To quantify changes in cell migration upon optoWnt stimulation, we performed single-cell tracking of optoWnt hESCs expressing a nuclear-localized mVenus (mVenus-NLS) fluorescent tag grown in 2D co-culture with WT hESCs (Fig. S8, Movies 4 and 5). During the first 24 h of illumination, optoWnt hESCs showed no change in cell migration speed, velocity or mean-squared displacement (MSD); however, within the ∼24-32 h time window, these metrics increased approximately twofold, confirming that optoWnt cells gain a migratory phenotype upon stimulation (Fig. 4E, Fig. S9). After ∼32 h of illumination, both the MSD and velocity were observed to plateau and then decline in response to aggregation of optoWnt cells, reflecting the increasing role of cellular confinement in determining migration dynamics. Indeed, these time scales align with the emergence of segregation after ∼40 h of illumination (Fig. 4C, Fig. S4D). The ratio of cell displacement over distance and the percentage of time a cell migrated without turning remained constant over time (Fig. S9D,I,J), indicating that optoWnt cells did not increase directionality of their migration. In summary, this live-cell imaging and tracking supports the conclusion that Wnt drives self-organization of differentiating mesendoderm and hESCs through changes in cell migration.

To confirm that self-organization was driven in an EMT-dependent manner, we performed genetic knockdown of EMT regulators in optoWnt hESCs. We found that knockdown of the key EMT transcription factor *SNAI1* (which encodes SNAIL protein) reduced the segregation capacity of optoWnt and WT co-cultures, whereas knockdown of *SNAI2* (which encodes SLUG protein) had no detectable effect (Fig. S10). Interestingly, these results mimic the phenotype of knockout mice deficient in *Snai1*, which die at gastrulation due to a failure to undergo EMT and mesoderm formation (Carver et al., 2001), in contrast to *Snai2*-deficient mice, which remain viable (Jiang et al., 1998). This finding suggests that EMT is required for the observed segregation.

To decouple the contribution of EMT-dependent cell migration versus cell state changes associated with EMT, such as differential cell adhesion, to the observed cell segregation, we perturbed co-cultures with small molecule inhibitors of migration. We treated co-cultures with blebbistatin, an inhibitor of myosin ATPase activity and thus actomyosin-based motility, and ROCK inhibitor (Y-27632), an inhibitor of RHO/ROCK-mediated actomyosin contractility and ECM adhesion necessary for cell migration. We found blebbistatin to be toxic to hESCs even at low doses, whereas ROCK inhibitor Y-27632 led to an altered phenotype of optoWnt aggregates (Fig. 4G,H). Specifically, although optoWnt and WT cells still segregated, treatment with Y-27632 resulted in smaller, more abundant, and more circular optoWnt aggregates that displayed a less distinct spatial separation at the boundary edges between the optoWnt and WT domains (Fig. 4I). This effect is consistent with local cell-cell interactions regulating aggregation without tissue-scale cell migration out of and within epithelial hESC colonies. These results suggest that cell migration is required for larger aggregate size and their distinct spatial separation, and that cell migration works in combination with cell state changes during differentiation and EMT to mediate segregation.

### Nodal/TGFβ signaling is required for optoWnt/WT co-culture self-organization

**Fig. 5.**
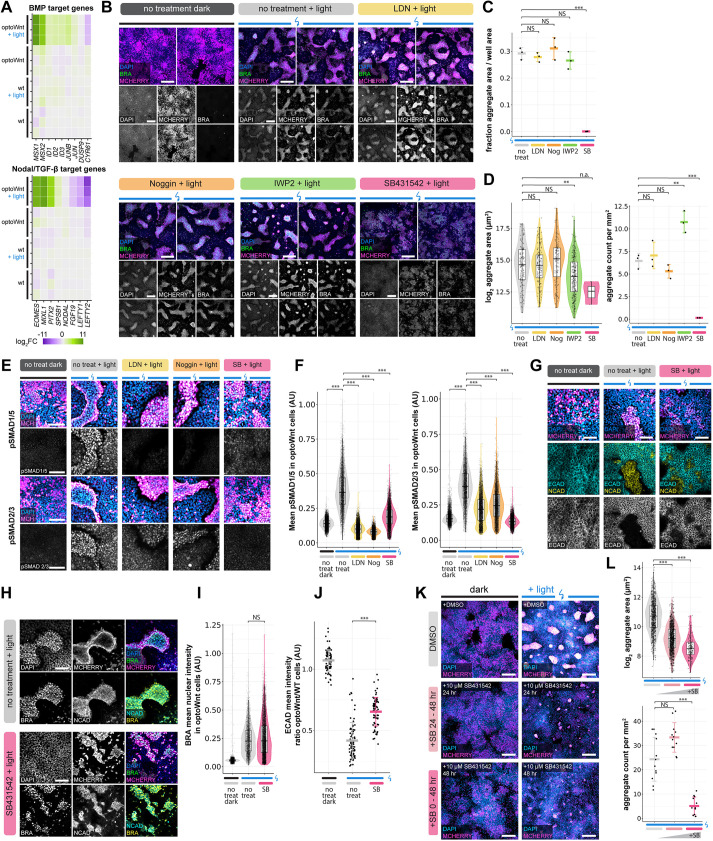
**Nodal/TGFβ signaling is required for optoWnt/WT co-culture self-organization.** (A) RNA-seq heat map of log_2_ fold change in BMP and Nodal/TGFβ target genes normalized to mean gene expression of WT hESCs. Biological replicates are displayed for each condition. (B) Representative images of immunostaining for mCherry (optoWnt), brachyury and DAPI in optoWnt/WT co-cultures under indicated inhibitor conditions after 48 h illumination. Two biological replicates are displayed. Scale bars: 250 µm. (C) Quantification of optoWnt aggregate area normalized to imaged area after 48 h illumination under no treatment or with indicated inhibitor treatments. Graph shows analysis of *n*=3 wells, mean±s.d, one-way ANOVA followed by Tukey's test (***P=8×10^−7^). (D) Quantification of optoWnt aggregate area (left, log_2_ transformed, **P_IWP2_=1.4×10^−5^) and number of aggregates per unit area (right, mean±s.d, ***P_SB_ 0.0001, **P_IWP2_=0.0023). Left graph uses Kruskal–Wallis test followed by pairwise Wilcoxon rank sum test with Bonferroni correction, and right graph uses one-way ANOVA with Tukey's test. (E) Representative images of immunostaining for phospho-SMAD1/5 (top) and phospho-SMAD2.3 (bottom), mCherry (optoWnt), and DAPI in optoWnt/WT co-cultures under indicated inhibitor conditions after 48 h illumination. Scale bars: 100 µm. (F) Quantification of mean phospho-SMAD intensity within optoWnt cells in optoWnt/WT co-cultures from immunostaining experiment shown in E. Each point represents the mean intensity of a nucleus. Kruskal–Wallis test followed by pairwise Wilcoxon rank sum test with Bonferroni correction (***P<10^−16^ for all). (G) Representative images of immunostaining for E-cadherin, N-cadherin, mCherry (optoWnt) and DAPI in optoWnt/WT co-cultures under indicated inhibitor conditions after 48 h illumination. E-cadherin image is reproduced below in grayscale for clarity. Scale bars: 100 µm. (H) Representative images of immunostaining for brachyury, N-cadherin, mCherry (optoWnt) and DAPI in optoWnt/WT co-cultures under indicated inhibitor conditions after 48 h illumination. Scale bars: 100 µm. (I) Quantification of brachyury nuclear intensity within optoWnt cells under indicated treatment from immunostaining experiment shown in H. Each point represents the mean intensity of a nucleus. Kruskal–Wallis test followed by pairwise Wilcoxon rank sum test with Bonferroni correction (*P*=0.32). (J) Quantification of E-cadherin intensity within optoWnt cells under indicated treatment from immunostaining experiment shown in G. Each point represents a field of view, where the mean E-cadherin intensity across optoWnt cells is divided by the mean E-cadherin intensity across WT cells in the same field. Kruskal–Wallis test followed by pairwise Wilcoxon rank sum test with Bonferroni correction (***P<10^−16^). (K) Representative images of immunostaining for mCherry (optoWnt) and DAPI in optoWnt/WT co-cultures treated with SB431542 inhibitor for the entire 48 h of culture or 24-48 h of culture (24 h duration) or untreated DMSO control. Scale bars: 250 µm. (L) Quantification of aggregate area (top) and number of aggregates per unit area (bottom) for experiment shown in K. Top: each point represents an aggregate area measurement (Kruskal–Wallis test followed by pairwise Wilcoxon rank sum test with Bonferroni correction, ***P<10^−16^ for all). Bottom: each point represents quantification of a field of view (one-way ANOVA followed by Tukey's test, *P*=0.10, ***P<0.0001). Box plots extend from 25th to 75th percentile; horizontal lines represent median; whiskers represent 1.5× interquartile range. The width of the shaded area (violin plot) represents the data density.

During mouse and human gastrulation, BMP and activin-Nodal-TGFβ signaling form feedback loops with Wnt signaling to reinforce mesendoderm differentiation and modify downstream fate outcomes (Brennan et al., 2001; Martyn et al., 2018; Massey et al., 2019). Consistent with this, we observed that target genes specific for both BMP and Nodal and/or TGFβ (Nodal/TGFβ) were activated in illuminated optoWnt cells (Fig. 5A). We thus sought to determine whether feedback from BMP, TGFβ and/or secreted Wnt ligand was necessary for cell segregation, or whether a sustained Wnt signal input was sufficient for segregation independent of downstream morphogen feedback. To this end, we treated co-cultures with the BMP receptor inhibitors noggin and LDN193189 (LDN), the Nodal/TGFβ receptor inhibitor SB431542 (SB), and the Wnt ligand secretion inhibitor IWP-2 (Fig. 5B). BMP receptor inhibition showed no observable effect on cell segregation or aggregate morphology, and Wnt secretion inhibition had similarly no observable effect on segregation but resulted in slightly smaller and more abundant optoWnt aggregates (Fig. 5B-D). Conversely, Nodal/TGFβ receptor inhibition showed a striking decrease in cell segregation, where optoWnt aggregates were barely detectable and had a significantly decreased abundance and size (Fig. 5C,D). Although there appeared to be a small amount of residual optoWnt clustering, SB treatment resulted in no distinct boundaries between WT and optoWnt domains, and no detectable optoWnt cell piling into vertical stacks (Fig. 5B, Fig. S5B).

Given the apparent necessity of Nodal/TGFβ signaling for cell segregation, we measured the levels of Nodal/TGFβ and BMP signal induction in optoWnt cells upon light stimulation of co-cultures, both in standard conditions and with inhibitor perturbation. We immunostained for the BMP and Nodal/TGFβ downstream effectors phospho-SMAD 1/5 (pSMAD1/5) and phospho-SMAD 2/3 (pSMAD2/3), respectively, (Galvin et al., 2010; Hollnagel et al., 1999) in co-cultures, and quantified expression at single-cell resolution (Fig. 5E,F). Consistent with target gene expression via RNA-seq (Fig. 5A), both pSMAD1/5 and pSMAD2/3 were upregulated in optoWnt cells after 48 h of light stimulation (Fig. 5E,F). We observed barely detectable pSMAD1/5 or pSMAD2/3 expression in unilluminated cultures, suggesting that induction occurred downstream of optoWnt stimulation, perhaps in the form of a Nodal/TGFβ autoregulatory feedback or increased cell responsiveness to TGFβ ligand in culture media. As expected, BMP inhibitor treatment reduced pSMAD1/5 in optoWnt cells to unilluminated levels, whereas Nodal/TGFβ inhibitor treatment reduced pSMAD2/3 (Fig. 5E,F). Thus, although illumination stimulated both SMAD1/5 and SMAD2/3 phosphorylation in optoWnt cells, only downregulation of pSMAD2/3 resulted in a decreased cell segregation phenotype.

We next sought to determine which cell processes could be mediating the observed effect of Nodal/TGFβ inhibition. We reasoned that the decrease in co-culture segregation under SB treatment may be due to its downstream inhibition of optoWnt cell differentiation, an inhibition of EMT and/or an inhibition of migration. We therefore quantified downstream effects of SB treatment on cell differentiation via BRA immunostaining, and EMT via E-cadherin and N-cadherin immunostaining (Fig. 5G-J). Interestingly, optoWnt cells were able to acquire BRA expression under SB treatment, with no difference in BRA nuclear intensity compared with untreated co-cultures (Fig. 5I). Furthermore, the switch in cell-adhesion proteins characteristic of EMT was maintained under SB treatment, with optoWnt cells showing a downregulation of E-cadherin and upregulation of N-cadherin (Fig. 5G,H). However, although E-cadherin expression decreased, optoWnt cells retained detectable expression in the SB treatment condition, suggesting an incomplete or delayed EMT (Fig. 5G,J). Indeed, changing the timing of SB treatment to the final 24 h of light stimulation (24-48 h) similarly abrogated segregation (Fig. 5K,L), suggesting that segregation can be inhibited even when cells have initiated differentiation and EMT. Instead, this 24-48 h period corresponds to the time window wherein optoWnt cells become most migratory (Fig. 4E,F), suggesting that TGFβ signaling feedback is necessary to maintain migration of optoWnt cells and to fully undergo EMT to ultimately enable cell segregation. In conclusion, segregation of co-cultures required both optoWnt activation and downstream Nodal/TGFβ signaling, which may play a role in maintaining EMT and optoWnt cell migration.

### Signaling from optoWnt mesendoderm cells induces TGFβ and BMP signaling in the WT epithelial subpopulation

**Fig. 6.**
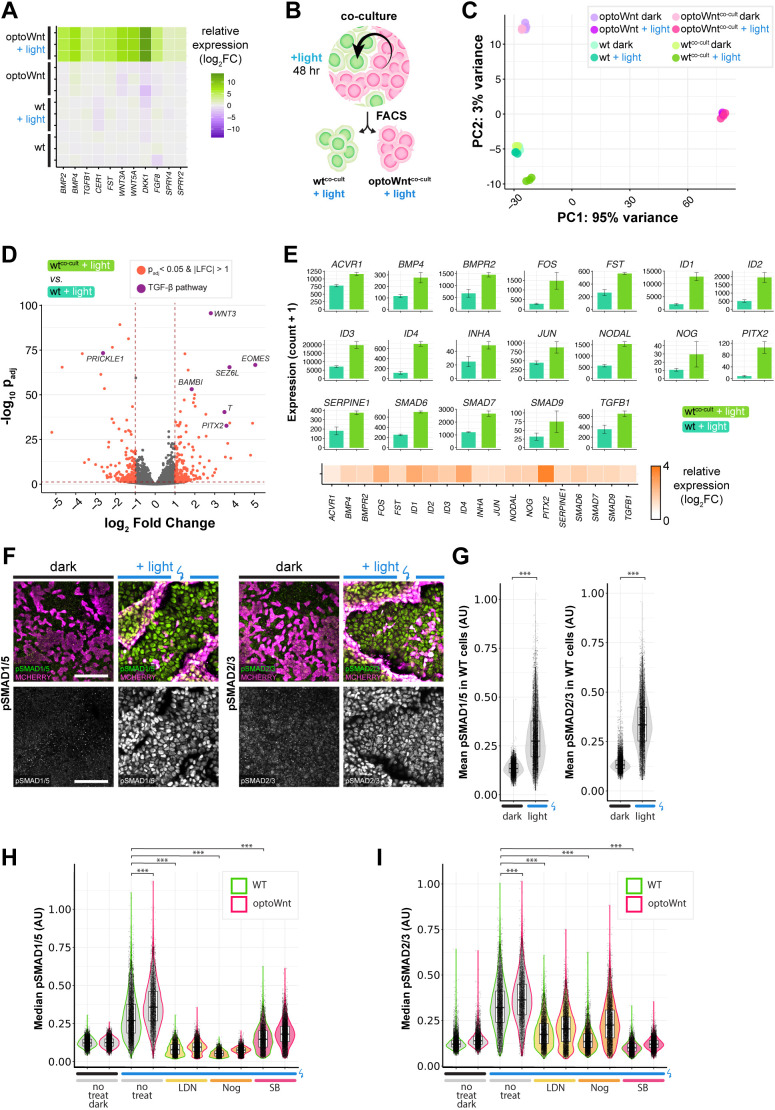
**Signaling from optoWnt mesendoderm cells induces TGFβ and BMP signaling in WT epithelial cells.** (A) RNA-seq heat map of log_2_ fold change in secreted morphogen genes, normalized to mean gene expression of WT hESCs. Biological replicates are displayed for each condition. (B) Schematic of FACS and RNA-seq experimental setup. (C) Principal component analysis (PCA) of RNA-seq results. Colors indicate the eight experimental conditions. Each point is a biological replicate. (D) Volcano plot of RNA-seq differential expression analysis of WT^cocult+light^ versus WT^light^ (monoculture) conditions, with DEGs (adjusted *P*-value<0.05 and log_2_ fold change>1) highlighted in red. Genes involved in the TGFβ pathway are highlighted in purple. (E) TGFβ pathway gene expression. Graphs show mean expression (read counts+1)±s.d., *n*=3 biological replicates. Bottom: log_2_ fold change in gene expression of indicated genes in WT^cocult+light^ versus WT^light^ (monoculture) conditions. (F) Representative immunofluorescence images of optoWnt/WT co-cultures stained for phospho-SMAD1/5 (left) and phospho-SMAD2/3 (right), with mCherry labelling optoWnt cells. Scale bars: 100 µm. (G) Quantification of mean phospho-SMAD intensity within WT cells in optoWnt/WT co-cultures from immunostaining experiment shown in F. Each point represents the mean fluorescence intensity of a nucleus. Unpaired two-sample Wilcoxon test (***P<10^−16^ for all). (H,I) Quantification of phospho-SMAD1/5 (H) and phospho-SMAD2/3 (I) intensity in both wild-type (green) and optoWnt (magenta) cells in optoWnt/WT co-cultures after 48 h illumination under indicated treatment conditions. Each point represents the median fluorescence intensity of a nucleus. Kruskal–Wallis test followed by pairwise Wilcoxon rank sum test with Bonferroni correction (***P<10^−16^ for all). Box plots extend from 25th to 75th percentile; horizontal lines represent median; whiskers represent 1.5× interquartile range. The width of the shaded area (violin plot) represents the data density.

Given the extensive gene expression changes in response to optoWnt stimulation, we hypothesized that the neighboring epithelial subpopulation of WT cells may receive paracrine signals from the differentiating mesendoderm population. Indeed, optoWnt-activated mesendoderm cells showed transcriptional upregulation of a variety of secreted morphogens, such as activators and inhibitors of Wnt, FGF, BMP and TGFβ signaling pathways (Fig. 6A). To determine which, if any, of these morphogens had a functional effect on the neighboring WT cell population, we performed RNA-sequencing to detect any transcriptional changes between hESCs grown in monoculture versus hESCs grown in co-culture with illuminated optoWnt cells (WT^cocult+light^). After 48 h of co-culture illumination, we performed fluorescence-activated cell sorting (FACS) to separate the optoWnt (mCherry^+^) and WT (GFP^+^) cell populations, and performed subsequent bulk RNA-seq analysis (Fig. 6B, Fig. S11A,B). PCA analysis showed clustering of biological triplicates for each condition and a notable shift of the WT^cocult+light^ population away from all other WT conditions (Fig. 6C). Gene expression analysis revealed that this WT^cocult+light^ population expressed genes such as *T*, *EOMES* and *BAMBI*, although expression levels of pluripotency markers *OCT4*, *SOX2* and *NANOG* remained unchanged (Fig. 6D, Fig. S11C). Further analysis of differentially expressed genes with pathway analysis software IPA and GSEA identified the TGFβ pathway as being strongly upregulated within the WT^cocult+light^ population (Fig. S11D,E), with accompanying strong upregulation of target genes such as *PITX2*, *NODAL* and *ID1*-*ID4* (Fig. 6E). At the protein level, immunostaining and single-cell quantification for phospho-SMAD1/5 and phospho-SMAD2/3 showed a strong upregulation only within WT epithelial cells that were in co-culture with illuminated optoWnt cells (Fig. 6F,G). Furthermore, this upregulation occurred throughout the epithelial colonies rather than being restricted to colony edges, suggesting that signaling was activated via a diffusible signal (Fig. 6F,G). Notably, mesendoderm cells did not yet induce differentiation of their epithelial neighbors after 48 h of illumination, as measured by retention of pluripotency markers (Fig. S11C), and there was no detectable effect on WT cell proliferation (Fig. S11F,G). These results suggest that the observed transcriptomic signature in the WT population could be mediated by BMP and TGFβ secretion from optoWnt mesendoderm cells, and/or by priming of WT cells to be more responsive to such signals from the cell culture medium.

We next sought to determine whether perturbation of BMP and Nodal/TGFβ receptors could reduce pSMAD signaling, which would support the hypothesis that the observed effect on WT cells is mediated at the receptor level. Treatment with BMP receptor inhibitors LDN and Noggin, and the Nodal/TGFβ receptor inhibitor SB showed strong reduction of pSMAD1/5 and pSMAD2/3, respectively, in WT cells, to levels comparable with unilluminated controls (Fig. 6H,I). Such signal inhibition at the receptor level suggests that WT cells display activated BMP/TGFβ signaling as a result of increased ligand availability or ligand responsiveness. In conclusion, after the emergence of organized mesendoderm patterns, TGFβ and BMP signaling was activated in epithelial neighbors, which may have *in vivo* relevance as a mechanism for priming epiblast cells for subsequent fate specification.

## DISCUSSION

Gastrulation is regulated by highly complex signaling, and investigating these processes in early epiblast and ESC-based models would benefit from the ability to perturb signaling in specific cell subpopulations, which can allow investigation of signaling mechanisms that control tissue-scale coordination. Once activated, Wnt signaling induces ESC differentiation and EMT (Davidson et al., 2012; Sumi et al., 2008; Turner et al., 2014), but how cell subpopulations may experience different Wnt signaling levels and how they, in turn, interact with one another during early development remains poorly understood. Here, we show that cell-to-cell variability of otherwise non-spatially patterned Wnt signaling is sufficient to induce cell self-organization and tissue-scale patterns. We thus establish an optogenetics-based model system to study early embryonic self-organization, and using single-cell tracking and transcriptomics analysis we show that organization occurs in an EMT- and TGFβ-dependent manner. Furthermore, Wnt-active cells form local signaling hubs to subsequently pattern neighboring epithelial populations.

Cell variability is emerging as an important regulator of tissue differentiation and developmental robustness (Losick and Desplan, 2008), and is particularly evident in pluripotent cell populations (Kumar et al., 2014; MacArthur and Lemischka, 2013). Targeting Wnt activation in cell subpopulations allowed us to model several key processes of early embryonic development. First, light stimulation of co-cultures mimicked the activation of Wnt signaling in the epiblast, which occurs before primitive streak formation (Peng et al., 2019; Rivera-Pérez and Magnuson, 2005; Tortelote et al., 2013; Yoon et al., 2015). With the hESC co-culture approach, we demonstrated that, in the absence of directional cues, such as gradients of Wnt signaling and morphogen secretion from extra-embryonic tissues, heterogeneous activation of Wnt is sufficient for self-organization and mesendoderm migration away from an epithelial cell population. Unlike classic Turing systems, cell-to-cell variability in WT/optoWnt co-cultures does not self-emerge but rather is directly controlled with optogenetic stimulation and leads to tissue-scale cell segregation and patterning. Consequently, emergence of cell-to-cell heterogeneity in Wnt signaling may contribute to tissue organization.

The finding that self-organization can occur in the absence of a localized source of Wnt signaling is consistent in general with modeling and experimental approaches. Indeed, cell-to-cell variability can naturally emerge from the intrinsic dynamics of cell systems and can lead to various tissue-scale spatial patterns, depending on the intercellular communication networks and reaction dynamics (Furusawa and Kaneko, 2012; Kondo and Miura, 2010; MacArthur et al., 2009; Turing, 1952). In particular, our findings shed light on the spontaneous self-organization of ESC gastruloid models (Beccari et al., 2018; Suppinger et al., 2023; ten Berge et al., 2008; Turner et al., 2017), where patterning could conceivably be influenced by heterogeneous Wnt pathway activation, e.g. due to variable cell sensitivity to Wnt treatment. Indeed, it has been reported that individual ESCs within a population show heterogeneous β-catenin activity (Blauwkamp et al., 2012; Davidson et al., 2012; Turner et al., 2014) and sensitivity to Wnt agonists (Davidson et al., 2012), potentially a result of geometric constraints and border effects (Blin et al., 2018; Muncie et al., 2020) and/or inherent variability in cell state and asymmetries in local cell environments (Kramer et al., 2022; Snijder et al., 2009; Suppinger et al., 2023). In an embryonic context, epiblast cell heterogeneity is similarly present before primitive streak formation (Mohammed et al., 2017; Scialdone et al., 2016) and may thus have a functional role in morphogenesis.

A second process that the optoWnt/WT co-culture system models is the emergence of a subpopulation of Bra-positive cells adjacent to a subpopulation of Bra-negative cells, mimicking the spatial positioning of cells at the primitive streak. Our model system thus allows studies of the signaling feedback, cell-cell interactions and dynamic cell rearrangement between these two populations. We observe an increase in TGFβ and BMP signaling in the epithelial cell population in response to inductive signals from the mesenchymal optoWnt population (Fig. 6). Such signal activation could serve to prime the epithelial population for differentiation toward specific lineages. For example, BMP4 has been shown to control ectoderm lineage specification in a graded manner: a strong pulse of BMP4 signaling is crucial for non-neural ectoderm derivation, whereas a low dose of BMP4 is optimal for neural crest and cranial placode differentiation, and no BMP4 with continuous dual SMAD inhibition results in neuroectoderm (Groves and LaBonne, 2014; Tchieu et al., 2017). We speculate that BMP/TGFβ signaling from the mesendoderm population could serve as a method for priming the epithelial subpopulation in a spatially coordinated way. Future studies with prolonged culture can investigate downstream cell fates of the epithelial subpopulation and correlate cell fate with BMP/TGFβ signaling dynamics.

Furthermore, optogenetic activation of Wnt signaling in hESCs models the process of EMT and allows the investigation of migration dynamics during EMT of the mesendoderm subpopulation. Single-cell migration analysis revealed heterogeneity in optoWnt migration velocity (0.1-0.3 µm/min) in response to optoWnt signaling (Fig. S9). In the mouse epiblast, cells display similarly heterogeneous migration speeds (McDole et al., 2018; Saykali et al., 2019), with temporal order of migration through the streak and cell displacement correlating with cell fate outcome (Kinder et al., 1999; Probst et al., 2021; Saykali et al., 2019). Although migration speed heterogeneity in our co-culture models is anticipated due to different local cell densities and confinement in 2D culture, an intriguing developmental analogy could be the spatial patterning of mesendodermal fates due to varying signaling dynamics across the primitive streak (Probst et al., 2021). The incomplete reduction of self-segregation under cell migration inhibitor treatment (Fig. 4G-I) suggests that, although cell motility is required for large-scale aggregation, motility works in combination with other cell state changes associated with differentiation and EMT, e.g. differential cell-cell adhesion (Steinberg and Takeichi, 1994), to mediate pattern shape and stability.

Finally, the optoWnt/WT co-culture system can be used to dissect and model signaling feedback mechanisms during hESC differentiation and EMT. The downstream activation of both Nodal/TGFβ and BMP signaling after optoWnt activation (Figs 4 and 5A) suggests that Wnt alone is sufficient for initiating the human BMP4-Wnt-Nodal primitive streak feedback loop without the need for exogenous BMP4 or activin stimulation (Ben-Haim et al., 2006; Chhabra et al., 2019; Etoc et al., 2016; Martyn et al., 2018; Simunovic et al., 2019; Warmflash et al., 2014; Xu et al., 2021), as observed in mouse *in vitro* models (Turner et al., 2017). Interestingly, Nodal/TGFβ signaling downstream of optoWnt stimulation is also necessary for the observed cell segregation (Fig. 5). This finding is consistent with genetic mutants of Nodal/TGFβ that are unable to form a primitive streak (Brennan et al., 2001; Dunn et al., 2004), inhibition of ESC differentiation via TGFβ or Wnt antagonist treatment (Gadue et al., 2006) and EMT processes during cancer metastasis that similarly require autocrine TGFβ signaling (Scheel et al., 2011). Our results show that this requirement for Nodal signaling can occur downstream of Wnt activation to regulate EMT progression and cell migration, suggesting a crosstalk between these two pathways during EMT. Indeed, SMAD proteins can associate with TCF/LEF proteins to mediate transcription (Labbé et al., 2000; Luo, 2017; Nishita et al., 2000), and a mechanistic study of Wnt/TGFβ synergy during primitive streak formation is a subject for future work.

In conclusion, we establish an experimental and conceptual framework for studying cell self-organization in early development through optogenetic control of cell-to-cell variability in Wnt signaling. As mechanisms underlying tissue-scale patterning remain poorly understood, this model system can be used for further studies of cell-cell interactions and intracellular regulation of cell polarity and migration. Furthermore, future approaches can be combined with cell position and light patterning techniques (Chen et al., 2016; Repina et al., 2020b, 2017) to control the relative positioning of cells and signaling dynamics, both in 2D and 3D cultures. Finally, optogenetic Wnt activation could be used for mechanistic analysis of Wnt signaling dynamics and spatiotemporal signaling thresholds in other systems, which would shed light on the ubiquitous nature of Wnt-mediated tissue patterning in developmental and stem cell biology.

## MATERIALS AND METHODS

### DNA vector assembly

All vectors were constructed using Gibson assembly or standard restriction enzyme cloning. To make all the AAVS1-Pur-CAG-optoWnt plasmids, AAVS1-Pur-CAG-EGFP (Addgene, 80945) was digested with SalI and MluI, and the Cry2-LRP6c construct containing the photolyase homology region of the *A. thaliana* Cry2 protein (Bugaj et al., 2013) was inserted with standard restriction enzyme cloning. To generate β-catenin luciferase reporter lines, the 7xTFP vector (Addgene, 24308) was modified to replace puromycin resistance with hygromycin resistance. To generate the Brachyury-2A-eGFP donor plasmid (Bao et al., 2019), DNA fragments of ∼2 kb in length were PCR amplified from the endogenous genomic *T* locus, before and after the stop codon, and were cloned into the OCT4-2A-eGFP donor plasmid (Addgene, 31938). Two sgRNAs targeting at or near the *T* stop codon (1, CACTGCATCTTTCGGGACCTGGG; 2, TGGCGACACAGGTGTCCATGAGG) were cloned into the CRISPR-GFP plasmid (Bao et al., 2016) via T4 ligation. To generate shRNA knockdown lines, shRNA sequences (Table S3) were subcloned into the pLKO.1 lentiviral expression vector digested with AgeI and EcoRI, and modified to express the blastocydin-resistance gene and eGFP.

### hESC cell culture

For routine culture and maintenance, all optogenetic and WT hESC lines (H9, WiCell) (Thomson et al., 1998) and iPSC lines (19-9-7, WiCell) (Yu et al., 2009) were grown on Matrigel (Corning, lot number 7268012, 7275006)-coated plates in mTeSR1 medium (STEMCELL Technologies) and 1% penicillin/streptomycin (Life Technologies) at 37°C and 5% CO_2_ with daily media changes. Optogenetic cells were cultured with hood lights off. For illumination experiments, cells were singularized with Accutase (STEMCELL Technologies) at 37°C for 5 min and seeded onto Matrigel-coated 24-well plates in media containing 5 µM ROCK inhibitor Y-27632 (Selleckchem). Cells were seeded at a density of 35-70k cell cm^−2^. For co-culture experiments, WT and optoWnt cells were mixed in a 1:1 ratio and seeded at a density of 35k cell cm^−2^. After 20-24 h, media were changed to E8 (STEMCELL Technologies), E6 (Thermo Fisher Scientific) or APEL 2 (STEMCELL Technologies) media without ROCK inhibitor, and plates were placed onto LAVA illumination devices and subjected to experimental conditions. When indicated, the Wnt agonist CHIR99021 (Stemgent) or recombinant Wnt3a protein (StemRD) was diluted in E8 media and added to cells.

Inhibitor treatments were performed with SB431542 (10 µM, Selleckchem), LDN193189 (100 nM, Selleckchem), recombinant Noggin (100 ng/ml, Peprotech), IWP-2 (5 µM, Selleckchem), ROCK inhibitor Y-27632 (10 µM, Selleckchem) and blebbistatin (5 µM, Selleckchem) diluted in E8 media. Unless otherwise indicated, cells were treated with inhibitor for 48 h during illumination.

### Generation of hESC and iPSC cell lines

Clonal knock-in lines were generated through CRISPR/Cas9-mediated recombination. Before nucleofection, hESCs were pre-treated with 10 µM ROCK inhibitor for 3-4 h or 5 µM Y-27632 overnight. Accutase-digested single hESCs were collected and 2.5-3.5 million cells were nucleofected with 2.5 µg gRNA AAVS1-T2 (Addgene, 41818), 4.5 µg pCas9-GFP (Addgene, 44719) and 6 µg optoWnt donor plasmid in 200 µl room temperature PBS (without calcium and magnesium) using a Nucleofector 2b (Lonza) with program B-016. The resulting cells were plated onto Matrigel-coated six-well plates containing 3 ml pre-warmed mTeSR1 with 10 µM ROCK inhibitor. Once the cells grew to confluency, they were subjected to selection with 1 µg ml^−1^ puromycin in mTeSR1 media for ∼2 weeks. Clonal lines were generated by picking single-cell clones into wells of a Matrigel-coated 96-well plate that were expanded for 1-2 weeks and subjected to PCR genotyping. As for the Bra-2A-eGFP reporter line, 3 µg T gRNA1, 3 µg T gRNA2 and 6 µg Bra-2A-eGFP donor plasmid were used. After the genotyping, targeted clones were treated with TAT-Cre to remove the PGK-PuroR cassette. Dual OptoWnt/Bra reporter lines were generated by CRISPR/Cas9-mediated knock-in of Cry2-LRP6c-2A-mCherry into the *AAVS1* locus of Bra eGFP reporter cells, as described above.

Knock-in at the *AAVS1* locus was verified through PCR on genomic DNA extracted with a Quick-DNA kit (D3024, Zymo Research). For the positive knock-in screen, a band size of 1.1 kb was expected using forward and reverse primers 5′ CTGTTTCCCCTTCCCAGGCAGGTCC and 5′ TCGTCGCGGGTGGCGAGGCGCACCG, respectively. For determination of zygocity, a band size of 0.2 kb was expected for heterozygous clones using forward and reverse primers 5′ CGGTTAATGTGGCTCTGGTT and 5′ GAGAGAGATGGCTCCAGGAA, respectively.

The β-catenin luciferase reporter lines were generated through lentiviral infection of WT and optoWnt hESCs with 7x TFP lentivirus packaged and purified from HEK 293T cells. Cells were infected with a multiplicity of infection of less than 1 and selected with 50-100 µg ml^−1^ hygromycin for 3 weeks.

Knockdown lines were generated by lentiviral infection of optoWnt hESCs with shRNA against target genes (Table S3). Infected cells were isolated by FACS sorting for eGFP expression. Knockdown was verified through western blot for target genes.

### Optogenetic stimulation

Cells were seeded on matrigel-coated 24-well plates (0030741021, Eppendorf, black-walled with 170 µm coverglass bottom) and placed onto LAVA illumination devices kept in standard 37°C tissue culture incubators (Repina et al., 2020a,b). In brief, user-defined illumination patterns were uploaded to the LAVA device for independent illumination control of each well. Unless otherwise noted, optogenetic stimulation was achieved with blue light emitted by arrays of 470 nm LEDs continuously illuminating hESCs with 0.8 µW mm^−2^ light for the duration of the experiment (1-48 h).

### Immunostaining and imaging

For 2D cell cultures, cells were fixed with 3% paraformaldehyde (Thermo Fisher Scientific) in PBS for 20 min at room temperature and subsequently washed three times with PBS, followed by blocking and permeabilization with 5% donkey serum (D9663, Sigma-Aldrich) and 0.3% Triton X-100 (Fisher Scientific) in PBS (PBS-DT) for 1 h. Cells were incubated with primary antibodies (Table S2) at 4°C overnight, then washed three times with PB, and incubated with fluorescently conjugated secondary antibodies (Invitrogen) at 1:250 dilution for 1 h at room temperature. Both primary and secondary antibodies were diluted in PBS-DT. Cells were washed with PBS and stained with 0.1 µg ml^−1^ DAPI nuclear stain (Thermo Fisher Scientific) before imaging.

Cell proliferation analysis with EdU was performed by treating cells with 10 µM EdU for 20 min before fixation. Staining for EdU was performed using a Click-iT EdU Alexa Fluor 647 kit (Invitrogen) following manufacturer specifications.

Confocal imaging was performed on a Perkin Elmer Opera Phenix system (QB3 High-Throughput Screening Facility). Bright-field and wide-field fluorescence imaging was performed on a Zeiss AxioObserver epi-fluorescent microscope and a Molecular Devices Image Xpress Micro imaging system (CIRM/QB3 Shared Stem Cell Facility).

### Live single-cell imaging and tracking

Co-cultures were treated with CellTracker Red (Thermo Fisher Scientific) dye diluted 5000× in mTeSR1 media for 15 min and washed twice. A sealing membrane (Breathe-Easy, Sigma-Aldrich) was applied to plates before imaging. Plates were imaged on a Molecular Devices Image Xpress Micro (IXM) imaging system with environmental control (37°C, 5% CO_2_ and humidity control) using a 10× objective. For each experiment, four to eight sites were imaged at 10-30 min intervals. For single-cell tracking experiments, cells were imaged at 18 min intervals. Optogenetic stimulation was delivered from the fluorescence light source (SOLA Light Engine, Lumencor) set to 5% intensity, passing through the 10× objective and GFP filter set (472/30 nm). Measured power at the sample was 2.82 mW. Optogenetic stimulation was delivered for 3 min at each site before imaging of each timepoint (i.e. for 3 min every 10-30 min) in a sequence of short light pulses (500 ms on-pulse, 10 s off-pulse).

Single-cell tracking analysis was performed as follows. To extract cell positions, the contrast on each image was corrected using adaptive histogram equalization, then the image background was removed using a difference of gaussians filter. Peaks at least 20% above background in the resulting foreground image were then detected using non-local maximum suppression (van der Walt et al., 2014) with a minimum radius of 2.6 µm. The cell counts were then fit to an exponential model of cell growth, and frames with anomalous (R^2^>2500 cells^2^) segmentations were discarded. This resulted in a dataset of individual cell detections in five fields of view spanning 90 frames between approximately hours 8 and 36 of the stimulation experiment.

Individual cell detections were linked to their nearest neighbor in a radius up to 32.4 µm from their previous position both forwards and backwards in time with both a global maximum velocity and neighborhood quasi-rigidity penalty (Kim and Lee, 2002). The resulting track fragments were then iteratively merged with overlapping tracks within 3.4 µm and 18 min of each other. This process converged after 10 iterations, generating 4127 total tracks with mean length of 13.5 h (standard deviation 6.4 h).

For each track, instantaneous velocity magnitude and direction were approximated using finite differences and then smoothed with a 15 point (4.5 h) rolling window filter. Cell migration distance was calculated by integrating over finite differences of cell position. Track turning angle was calculated by a phase unwrapping change in velocity direction. Finally, periods of persistent migration were determined as those times where a cell was both migrating at least 0.1 µm/min and turning no more than 2 degrees/min from its previous velocity direction. Both changes in instantaneous traces and binned values were assessed based on 95% confidence intervals around the mean, as determined by 1000 iterations of bootstrap sampling.

### Image analysis

Microscopy image processing, including stitching and z-slice projection, was performed in Fiji (Schindelin et al., 2012); image quantification was performed in CellProfiler (Carpenter et al., 2006) with custom analysis pipelines detailed below.

For nuclear detection, nuclei stained with DAPI were segmented with CellProfiler via Otsu thresholding. The generated label mask was applied to the other fluorescence channels for feature extraction. The mean β-catenin intensity per cell nucleus was calculated for each cell in a given field of view. A similar quantification strategy was adopted for quantification of the percentage positive cells for a given nuclear-localized cell fate marker (e.g. BRA, SOX2, OCT4 and NANOG) with a threshold defining ‘positive’ cells determined from signal intensity of negative controls (see Fig. S2).

Quantification of cell segregation in 2D cultures was performed as follows. Nuclei were segmented as above based on the DAPI channel images. Each nucleus was then classified as either mCherry positive or negative based on its mean mCherry nuclear intensity. For each mCherry-positive (mCh^+^) nucleus, the number of total nuclei and the number of mCh^+^ nuclei that neighbored the cell nucleus within a 15 µm expanded perimeter was calculated using the MeasureObjectNeighbors module in CellProfiler. The distribution of total neighbors versus mCh^+^ neighbors was then plotted directly and was also used to calculate the neighbor fraction (i.e. mCh^+^ neighbors/total neighbors) for each mCh^+^ nucleus. Next, for each field of view (16-54 per biological replicate), the mCh^+^ cells whose neighbors were all mCh^+^ (i.e. neighbor fraction=1) and that had more than two neighbors were counted and normalized to the total number of mCh^+^ cells in the field of view. This value, the fraction mCh^+^ cells with all mCh^+^ neighbors, was used as a metric for quantifying cell segregation. In a well-mixed population, a small fraction of cells should have a neighbor fraction equal to 1, whereas in a perfectly segregated population, all cells except those located at boundary edges should have a neighbor fraction equal to 1.

For quantification of aggregate area and count, aggregates were manually segmented in Fiji based on DAPI and mCherry channels, and the area and count statistics per field were normalized to field area. Feature measurements such as aggregate area and circularity were extracted using default Fiji measurement functions.

For single-cell SMAD, ECAD and BRA expression quantification of co-cultures, the mean or median fluorescence intensity per nucleus was measured in nuclei that were classified as mCh^+^ (for optoWnt cells) or mCh^−^ (for WT cells) using CellProfiler as described above.

### Luciferase assay

Wild-type and β-catenin reporter hESCs were rinsed with PBS, lysed with lysis buffer (E1531, Promega) and centrifuged to pellet debris. Firefly luciferase expression was quantified by adding 100 µl Luciferase Assay Reagent (E1500, Promega) to wells of an opaque 96-well plate containing 20 µl lysate supernatant, with resulting luminescence immediately detected on a luminometer (SpectraMax, Molecular Devices). Luminescence intensity was normalized to total protein concentration (Pierce BCA protein assay, Thermo Fisher Scientific) to account for proliferation or seeding density variability between samples.

### Flow cytometry and analysis

Cells were lifted with Accutase at 37°C for 5 min, centrifuged and resuspended in flow buffer [0.5% bovine serum albumin in PBS (w/v)] for analysis. Flow analysis and FACS sorting was performed on a BD Fortessa X20 or BD Aria cell sorter, respectively (CRL Flow Cytometry Facility). Cell counting for proliferation assays was performed using a Thermo Fisher Scientific Attune (CIRM/QB3 Shared Stem Cell Facility) by measuring the number of cells per unit volume.

Data analysis was performed with FlowJo 10 software. To determine the fraction of BRA-eGFP+ cells after light treatment, gating was set such that less than 0.5% of undifferentiated WT hESCs were positive for eGFP or mCherry.

### RT-qPCR

Cells were lifted with Accutase at 37°C for 5 min, centrifuged and resuspended in TRI reagent (Zymo Research). To achieve higher RNA yields, two or three wells were pooled, constituting a single biological replicate. RNA was purified using an RNA extraction kit (R2051, Zymo Research) as per the manufacturer's recommendations with an on-column DNase digestion to remove residual genomic DNA. After measurement of total RNA concentration, 1 µg of RNA was converted to cDNA using an iScript cDNA synthesis kit (Bio-Rad). Finally, 10 ng of cDNA was used for each SYBR Green qPCR reaction, run in 96-well plate format with a 0.1 µM final forward and reverse primer concentration (Table S3). qPCR was conducted for 50 cycles at an annealing temperature of 56°C on a CFX Connect Real-Time PCR Detection System (Bio-Rad). A melt curve was generated at the end of the PCR reaction and a subset of reactions were run on a 1% agarose gel to ensure that only one product of the expected size was amplified per primer pair. qPCR analysis was conducted by the ddC_t_ method. For each cDNA sample, gene expression was internally normalized to the expression of a human housekeeping gene (RPS18) run on the same qPCR plate. Next, for each gene, expression was normalized to the expression level of WT untreated hESCs. The log of relative expression over this WT control [i.e. log_2_(fold change)] was graphed as a heatmap where color corresponds to the mean value of biological replicates. The variability in gene expression was assessed with histogram graphs that show mean and standard deviation of fold change for three biological replicates, with at least two technical replicates for each biological replicate.

### RNA-sequencing and data analysis

For RNA-sequencing of co-cultured WT and optoWnt cells, FACS (as described above) was performed before RNA purification to separate the two populations based on GFP and mCherry signal, respectively. For gating strategy, see Fig. S11. Sorted samples were immediately centrifuged and resuspended in TRI reagent, and RNA was purified as described above (R2051, Zymo Research), with three biological replicates per experimental condition, and stored at −80°C until library preparation.

Library preparation, sequencing and data pre-processing were performed by MedGenome (Foster City, CA, USA). In short, libraries were generated with the TruSeq Stranded mRNA Library Prep Kit (Illumina) and sequencing was performed on an Illumina HiSeq 4000 with a sequencing depth of ∼35-65 million aligned reads per sample. The quality of reads was evaluated using the FASTQC tool. Adapter trimming was performed using Fastq-mcf and Cutadapt, followed by contamination removal using Bowtie2. Paired-end reads were aligned to the reference human genome (GRCh38, Ensemble database) using STAR alignment software and raw read counts were estimated using HTSeq. Normalization and differential expression analysis were performed in DESeq2 (Love et al., 2014). Genes with an adjusted *P*-value less than 0.05 and fold change greater than 2 were identified as differentially expressed genes (DEGs). PCA analysis, heatmaps and volcano plots were generated in R. Unless otherwise stated, fold change in heatmaps was normalized to the WT unilluminated control and calculated using normalized read count+1 values.

### Western blotting

Cells were lysed in RIPA buffer (Sigma) with phosphatase and protease inhibitors (EMD Millipore). Protein content was measured by bicinchoninic acid (BCA) assay and used to normalize samples to the lowest concentration. Lysates were heated to 95°C and run on a 4-12% Bis-Tris gel (Life Technologies) and transferred to a nitrocellulose membrane. Membranes were blocked in Odyssey blocking buffer (Li-Cor) and incubated with primary antibody at 4°C overnight (Table S2). All membrane wash steps were performed using Tris-buffered saline with 0.1% Tween-20. Membranes were incubated with secondary antibody, IRDye 800 goat anti-mouse IgG and IRDye 700 goat anti-rabbit IgG (Li-Cor) at a 1:10,000 dilution in blocking buffer for 1 h at room temperature. Blot fluorescence was visualized using an Odyssey CLx system (Li-Cor) and quantified with the built-in gel analyzer tool in ImageJ.

### Statistical analysis and graphing

Data are presented as mean±s.d. unless otherwise specified. Statistical significance was determined using an unpaired Student's *t*-test (two-tail) between two groups, and three or more groups were analyzed by one-way analysis of variance (ANOVA) followed by Tukey's test. The unpaired two-sample Wilcoxon test was used for two-sample data that was not normally distributed, and the Kruskal–Wallis test followed by a pairwise Wilcoxon rank sum test with Bonferroni correction was performed to calculate pairwise comparisons between group levels for multi-group data that was not normally distributed. *P*<0.05 was considered statistically significant (NS, *P*>0.05; *P<0.05; **P<0.01; ***P<0.001). Statistical analysis and data plotting were performed in R.

## Supplementary Material

10.1242/develop.201386_sup1 Supplementary information
