# Supplementary material for: Optogenetic control of Wnt signaling models cell-intrinsic embryogenic patterning using 2D human pluripotent stem cell culture
Source: Development. 2023 Jul 26;150(14):dev201386. doi: 10.1242/dev.201386 (PMC10399980; doi:10.1242/dev.201386)
Supplement: Supplementary information [file develop-150-201386-s1.pdf]

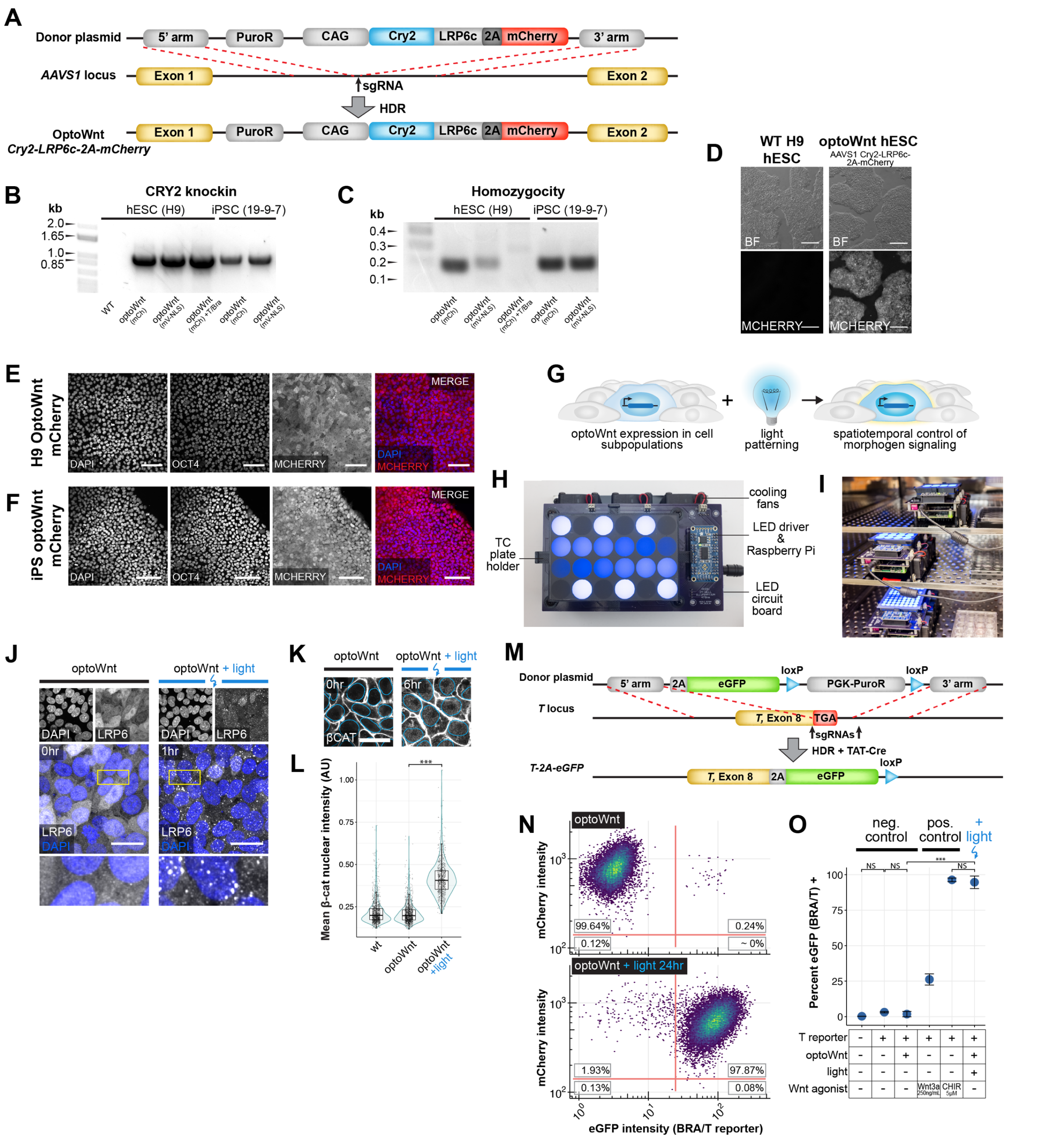

Fig. S1. OptoWnt cell line characterization and LAVA optical stimulation setup
a) Schematic of optoWnt knock-in strategy using CRISPR-mediated modification of *AAVS1* locus. OptoWnt expression driven by synthetic CAG promoter. **b)** PCR genotyping of hESC and iPSC clones after puromycin selection. Expected PCR product for correctly targeted *AAVS1* locus is 1.1 kbp. **c)** PCR homozygosity assay on knock-in clones. Clones without a ~200 bp PCR product were homozygous. **d)** Representative brightfield (BF) and mCherry fluorescence images of live WT and optoWnt hESCs. Scale bar 250 μm. **e)** Representative images of immunostaining for OCT4 and mCherry in optoWnt hESCs in routine cell culture, kept in the dark. Scale bar 100 μm. **f)** Representative images of immunostaining for OCT4 and mCherry in optoWnt iPSCs in routine cell culture, kept in the dark. Scale bar 100 μm. **g)** Schematic of optogenetic experimental setup for stimulation of cell subpopulations. **h)** Image of illumination device, LAVA board, used for optogenetic stimulation of hESC cultures. Blue light-emitting

diodes (LEDs) illuminate a tissue culture (TC) plate placed onto LAVA board. **i)** Image of LAVA boards kept inside a TC incubator. **j)** Immunostaining for LRP6 in optoWnt hESCs in the dark (left) and after 1 hr of 0.8 μW mm^{-2} illumination (right). Scale bar 25 μm. **k)** Representative images of immunostaining for β-catenin in optoWnt hESCs in the dark (left) and after 6 hr illumination (right). Nuclear outline from DAPI stain overlaid in blue. Scale bar 25 μm. **l)** Quantification of β-catenin nuclear intensity shown in (k). Graph shows pooled analysis of 14 fields of view per biological replicate (n = 3), each point representing a single cell. Unpaired two-samples Wilcoxon test ($p < 10^{-16}$). **m)** Schematic of eGFP knock-in strategy to make BRA/T reporter line using CRISPR-mediated modification of endogenous BRA/T locus. **n)** FACS analysis of optoWnt hESCs (mCherry+) modified with an eGFP reporter at the endogenous BRA/T gene locus ("BRA/T reporter"), kept in the dark (top) or illuminated for 24 hrs (bottom). Graph shows pooled data from 3 biological replicates, ~30,000 cells per condition. **o)** FACS quantification of percent eGFP-positive cells under indicated conditions, treated for 24 hrs, e.g. Wnt3a recombinant protein (250 ng/mL), CHIR (5 μM), or light stimulation (0.8 μW mm^{-2}). ANOVA followed by Tukey test ($p < 10^{-12}$). Graph shows mean ± 1 s.d., n = 3 to 6 biological replicates.

Development • Supplementary information

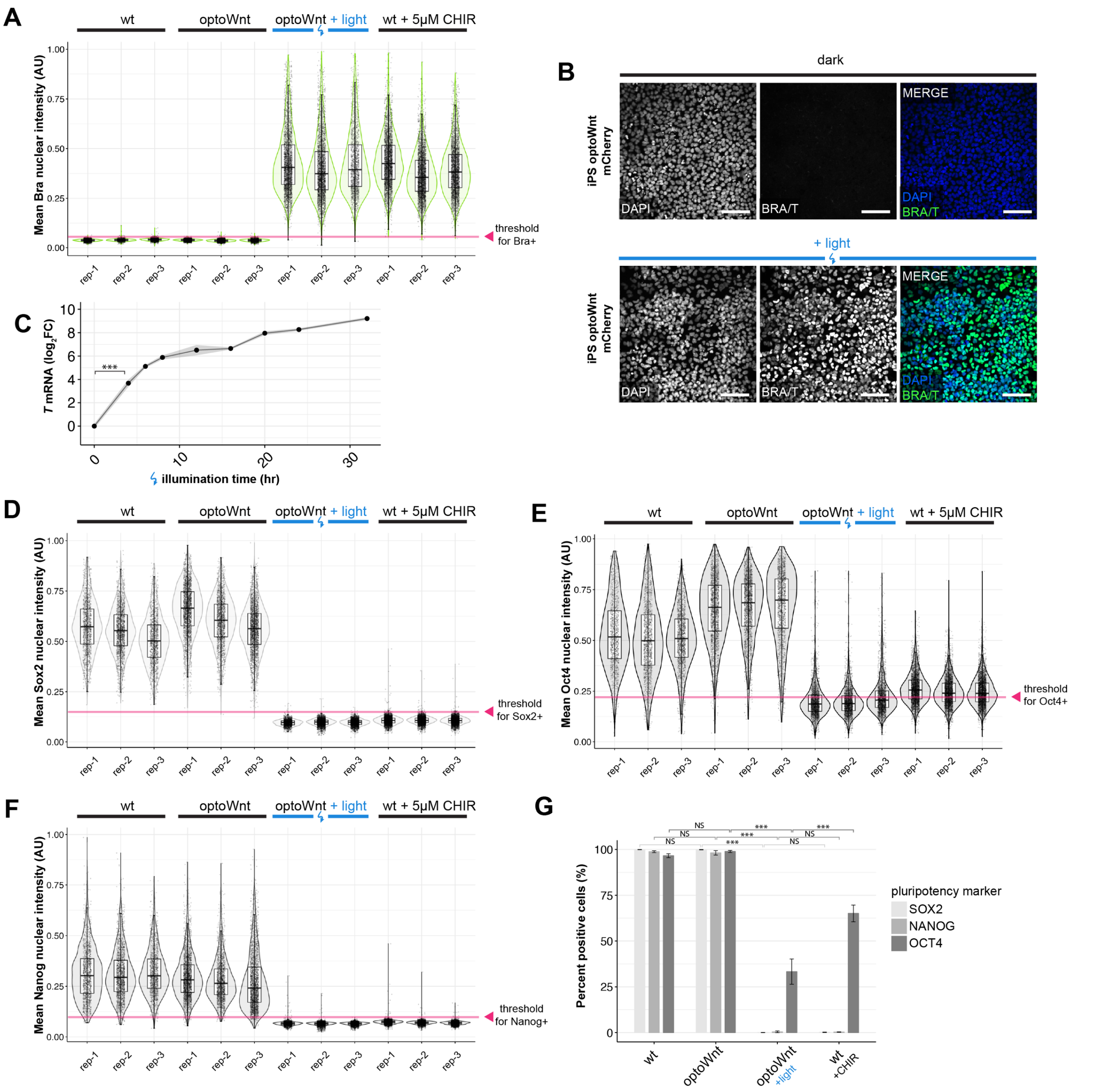

Fig. S2. Lineage marker expression in optoWnt hESCs and iPSCs

a) Quantification of mean BRA nuclear intensity from immunostaining shown in Figure 1C. Graph shows analysis of biological replicates (rep 1-3) after 48 hrs illumination or CHIR99021 (5 μM) treatment. Each point represents a single cell. Threshold for BRA+ classification indicated with red arrow. **b)** Representative images of immunostaining for BRA in optoWnt iPSCs in the dark (top) or after 48 hrs illumination (bottom). Scale bar 100 μm. **c)** qPCR timecourse of *T* mRNA expression in optoWnt hESCs at indicated durations of illumination. Graph shows mean log fold change in mRNA expression (log_2FC) relative to dark (0 hr) condition ± 1 S.E.M, n = 3 biological replicates. **d-f)** Quantification of mean SOX2 (d), OCT4 (e), and NANOG (f) nuclear intensity from immunostaining shown in Figure 1E. Graphs show analysis of biological replicates (rep 1-3) after 48 hrs illumination or CHIR99021 (5 μM) treatment. Each point represents a single cell. Threshold for SOX2+, OCT4+, or NANOG+ classification indicated with red arrow. **g)** Quantification of percent positive cells for pluripotency markers based on immunostaining shown in (d)-(f) and Figure 1E. Graph shows percent positive cells ± 1 s.d., n = 3 biological replicates. ANOVA followed by Tukey test.

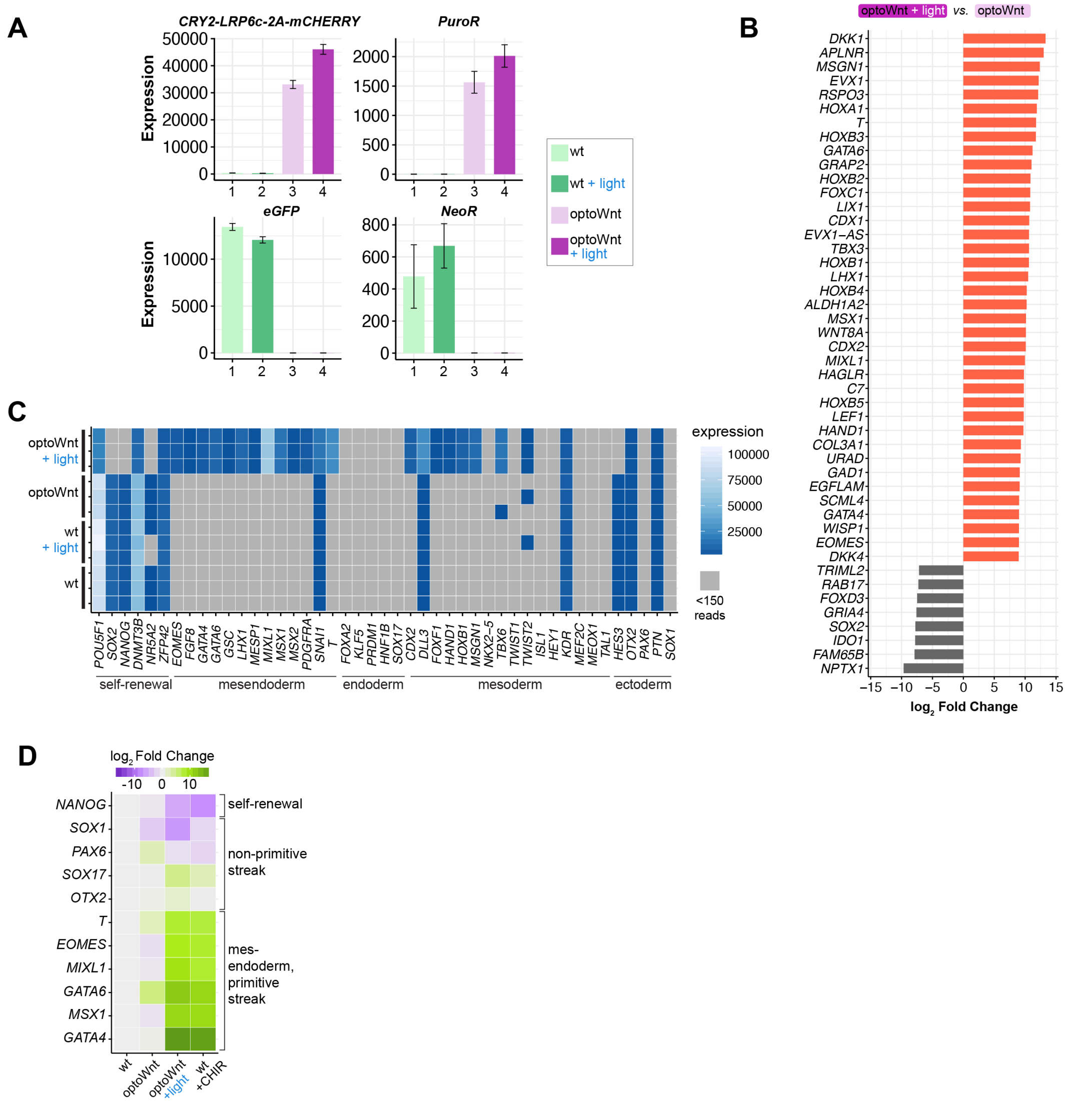

Fig. S3. Validation of RNA-seq study of optoWnt-induced hESC differentiation

a) RNA-seq results of indicated genes that mark WT (eGFP+, Neomycin-resistant) and optoWnt (Cry-LRP6c-2A-mCherry+, Puromycin-resistant) cells. Graphs show mean expression (read count + 1) ± 1 s.d., n = 3 biological replicates. **b)** Top upregulated (red) and downregulated (grey) genes in illuminated optoWnt vs. unilluminated optoWnt hESCs. Graph shows mean log2 fold change for each indicated gene. **c)** Heat map of mRNA expression (read count + 1) of indicated lineage markers. Biological replicates displayed for each condition, with undetected genes (read count < 150) shown in grey. **d)** Heat map of mean log2 fold change in lineage markers normalized to WT expression level from qPCR validation of RNA-seq results with indicated lineage markers and comparison to CHIR (3 μM) treatment for 48 hrs. Heatmap shows mean fold change of n = 3 biological replicates.

Development • Supplementary information

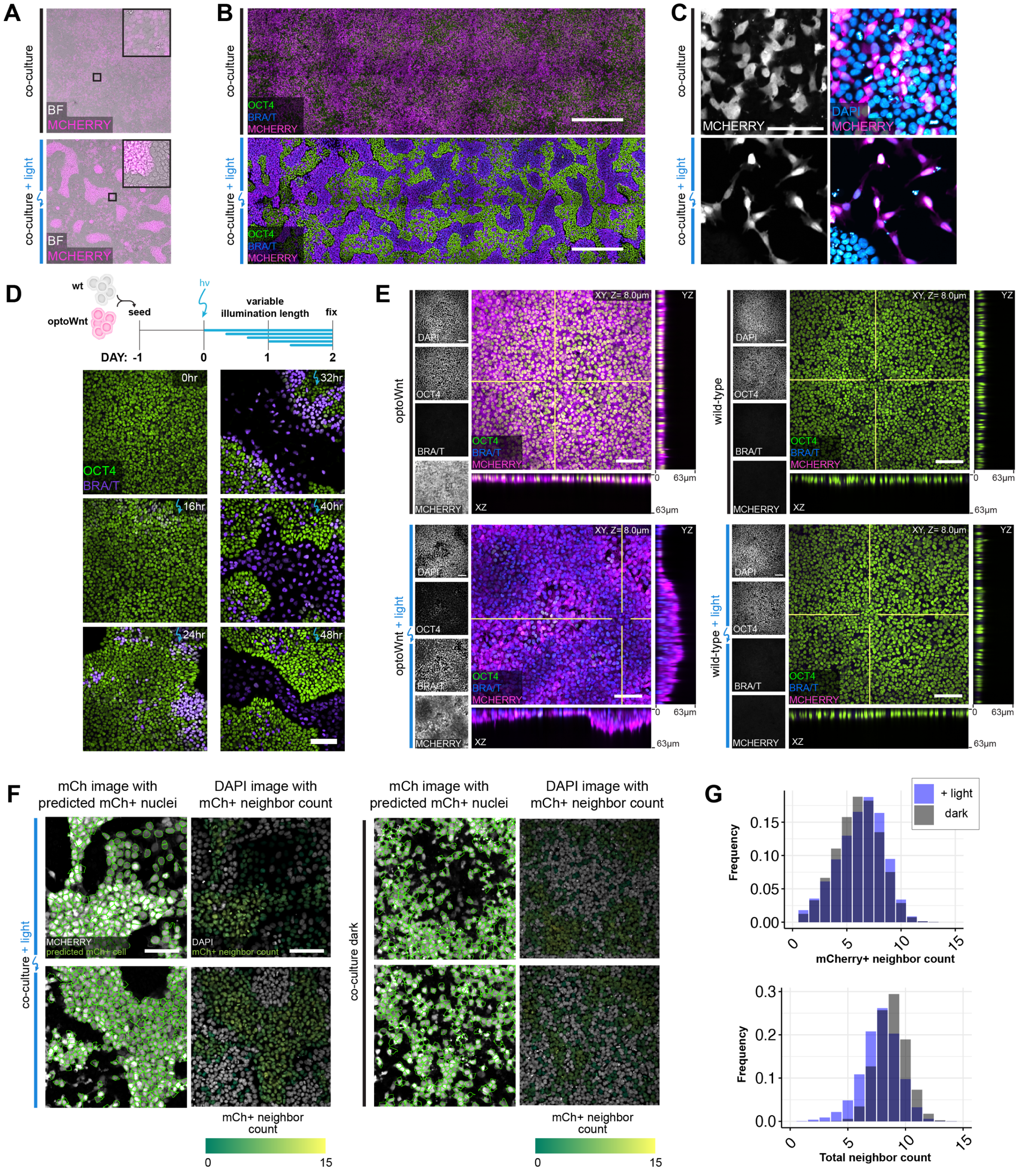

Fig. S4. Quantification of cell self-organization upon optoWnt stimulation of cell subpopulations

a) Representative images of brightfield (BF) and mCherry fluorescence (expressed in optoWnt cells) of fixed optoWnt/WT co-cultures in the dark (top panel) after 48 hrs of illumination (bottom panel). Scale bar 100 μm. **b)** Stitched images of optoWnt/WT co-cultures show large-scale pattern of cell self-organization. Scale bar 500 μm. **c)** Representative image of optoWnt/WT co-cultures seeded at lower cell density (20k cell cm^{-2}) show single-cell scattering and morphology changes in optoWnt (mCh+) cells after 48 hrs illumination. Scale bar 100 μm. **d)** Representative fluorescence images of optoWnt/WT co-cultures after

indicated durations of illumination, stained for OCT and BRA/T. Scale bar 100 μm. **e)** Confocal images of optoWnt monocultures (left panels) and WT monocultures (right panels) in the dark or after 48 hrs illumination, stained for OCT4 and BRA/T. OptoWnt cells labelled with mCh. Scale bar 100 μm, YZ and XZ axial cross-sections shown through indicated slices (white lines), 63 μm in height. **f)** Sample images of cell neighbor analysis in CellProfiler of illuminated (left panel) and dark (right panel) co-cultures. Nuclear outline of mCh+ nuclei (green) overlaid on mCh channel image (left column). DAPI channel image (right column) shown with overlay of mCh+ nuclei colored by mCh+ positive neighbor count. Scale bar 100 μm. **g)** Histogram of total cell neighbor counts (bottom) and mCh+ cell neighbor counts (top) across all analyzed cells show comparable cell densities between light and dark conditions.

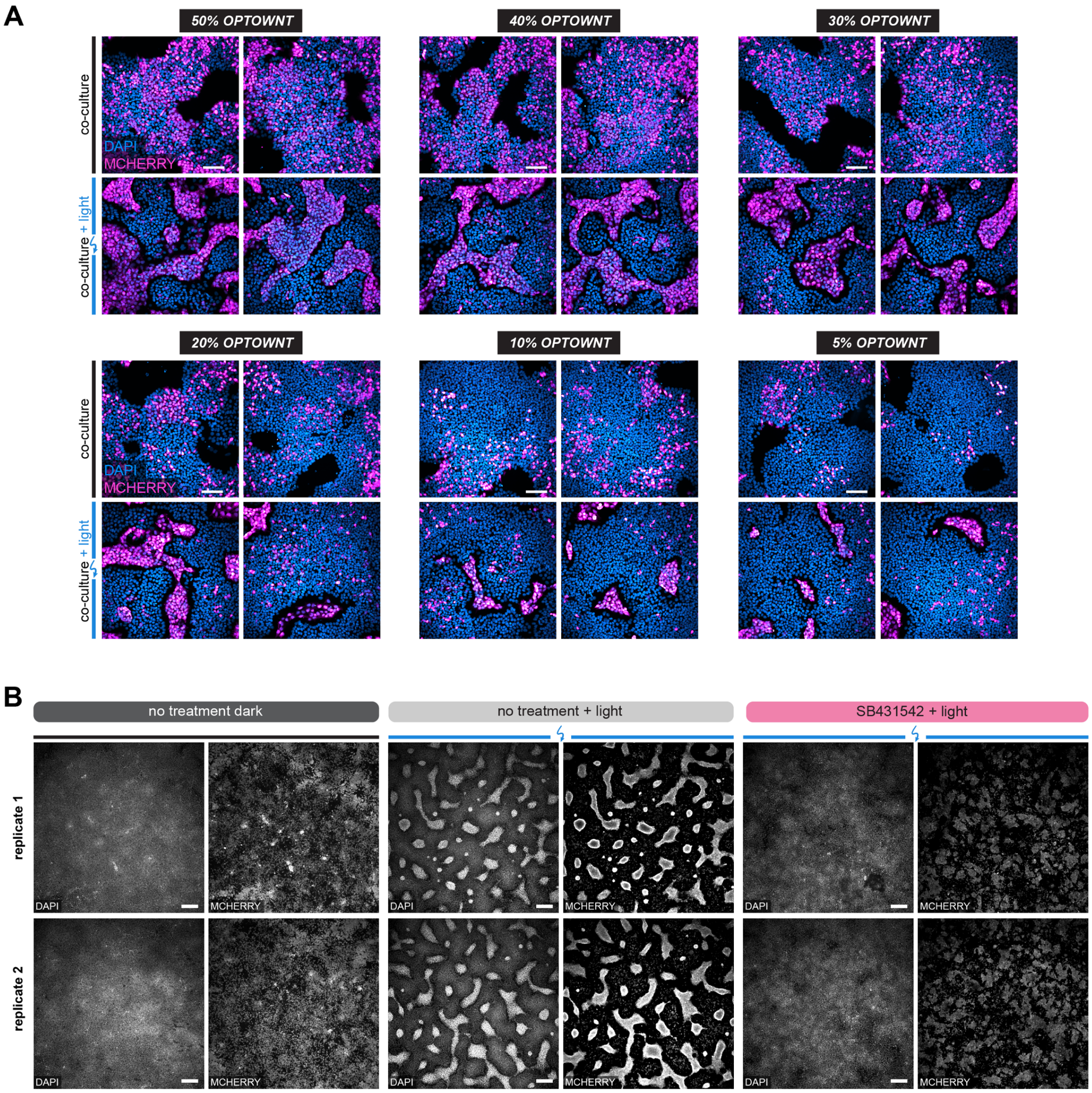

Fig. S5. Cell self-organization at variable dosages of optoWnt cells and SB inhibitor treatment in optoWnt/WT co-cultures.

a) Representative fluorescence images of optoWnt/WT co-cultures at indicated seeding doses (e.g. 40% optoWnt indicates 2:3 ratio of optoWnt:WT cells). OptoWnt cells are mCh+. Scale bar 100 μm. **b)** Representative fluorescence images of optoWnt/WT co-cultures under SB431542 inhibitor treatment, with two biological replicates displayed. Scale bar 250 μm.

Development • Supplementary information

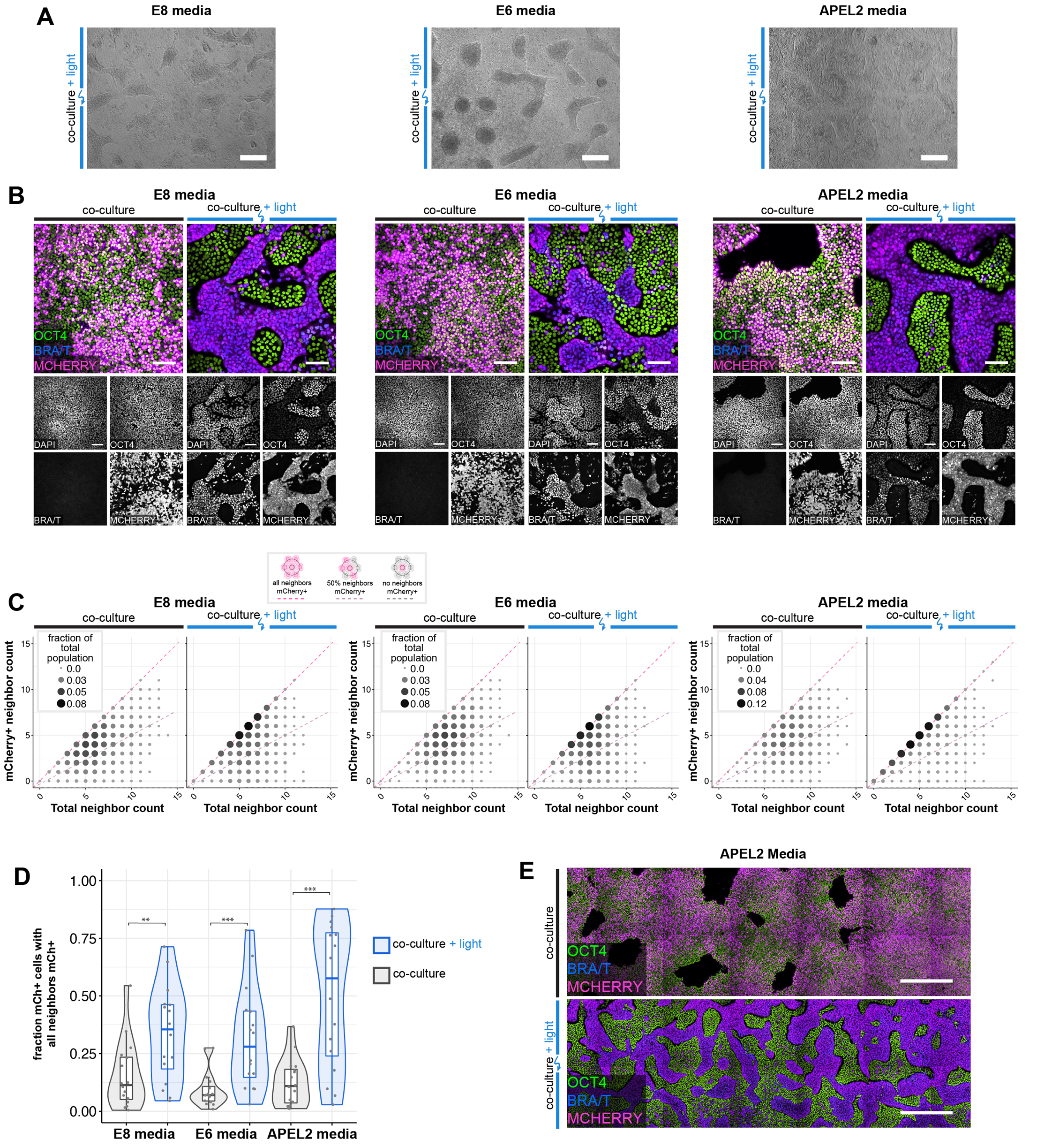

Fig. S6. Cell self-organization occurs in media without FGF and TGF-β agonists.

a) Representative brightfield images of optoWnt/WT co-cultures after 48 hrs illumination in E8, E6, and APEL2 media. Scale bar 250 μm. **b)** Confocal images of optoWnt/WT co-cultures in indicated media conditions, stained for OCT4 and BRA/T. OptoWnt cells labelled with mCh. Scale bar 100 μm. **c)** Cell neighbor analysis of optoWnt (mCh+) cells in co-culture. Graph shows count of total cell neighbors vs. count of mCh+ cell neighbors across total population of analyzed mCh+ cells (25,742 cells analyzed, pooled analysis from n=3 biological replicates for each condition). Area and color of points is proportional to the fraction of total population. Constant ratios of mCh+ to total neighbors are highlighted with dotted lines as indicated. **d)** Quantification of fraction of optoWnt (mCh+) cells whose neighbors are all mCh+. Each point represents an analyzed field of view (16 fields of view analyzed per condition, n = 3 biological replicates). Unpaired two-samples Wilcoxon test ($p_{E8} = 0.011$; $p_{E6} = 3.3 \times 10^{-4}$; $p_{APEL2} = 3.9 \times 10^{-4}$). **e)** Stitched images of optoWnt/WT co-cultures in APEL2 media show large-scale pattern of cell self-organization. Scale bar 500 μm.

Development • Supplementary information

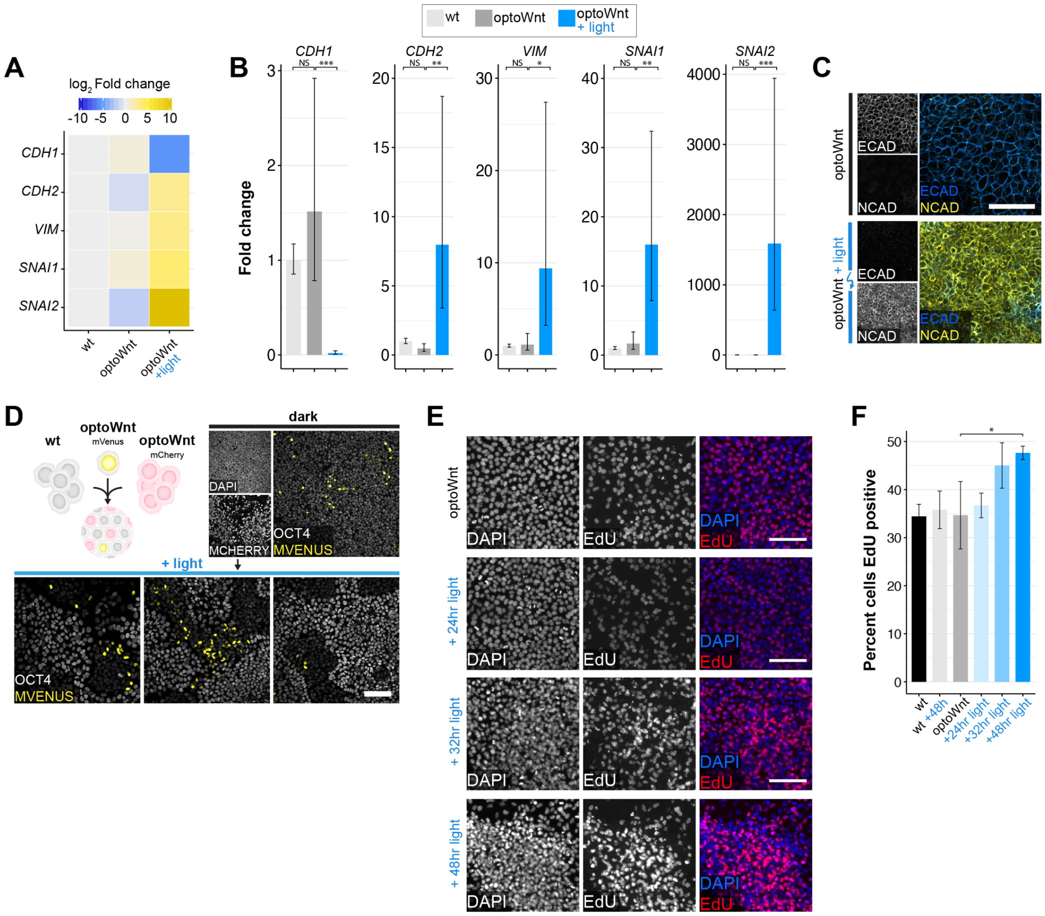

Fig. S7. EMT and increased cell proliferation upon optoWnt stimulation

a-b) qPCR for EMT markers in WT and optoWnt hESC monocultures cultured in APEL2 media. Graphs show heatmap of mean $\log_2$ fold change (a) and mean fold change (b) over WT hESCs ± 1 s.d., n = 3 biological replicates. ANOVA followed by Tukey test. **c)** Representative images of immunostaining for E-cadherin (ECAD) and N-cadherin (NCAD) in unilluminated (top) and illuminated (bottom) optoWnt cells. Scale bar 100 μm. **d)** No observed clonal expansion of optoWnt cells when optoWnt-mVenus-NLS cells were dosed into optoWnt-mCh/WT co-cultures. Scale bar 100 μm. **e-f)** EdU stain of optoWnt monocultures after indicated illumination duration, with representative images of EdU staining (e) and quantification of percent EdU+ cells (f). Graph shows mean ± 1 s.d., n=3 replicates. ANOVA followed by Tukey test. Scale bar 100 μm.

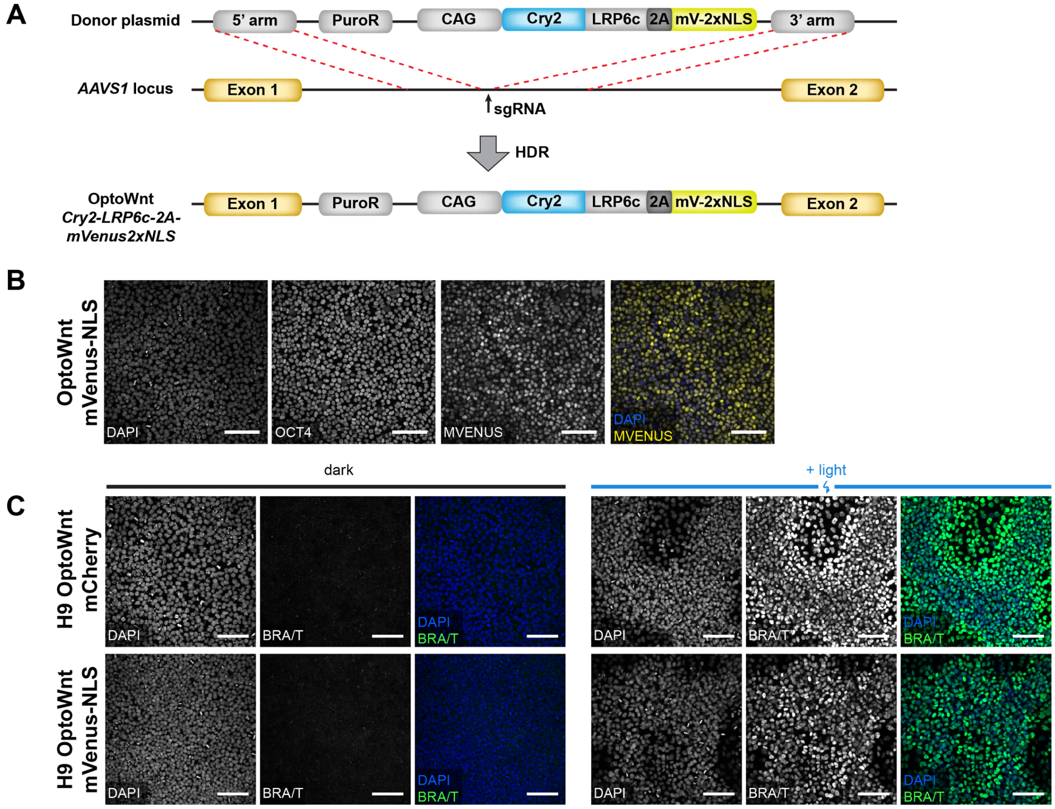

Fig. S8. Characterization of optoWnt-mVenus-NLS hESC line.

a) Schematic diagram of knock-in strategy at the *AAVS1* locus. **b)** Representative images of immunostaining for OCT4 and mVenus in optoWnt-mVenus-NLS hESCs in routine cell culture, kept in the dark. Scale bar 100 μm. **c)** Light-induced BRA expression of optoWnt-mVenus-NLS line is comparable to optoWnt-mCherry line. Scale bars 100 μm.

Development • Supplementary information

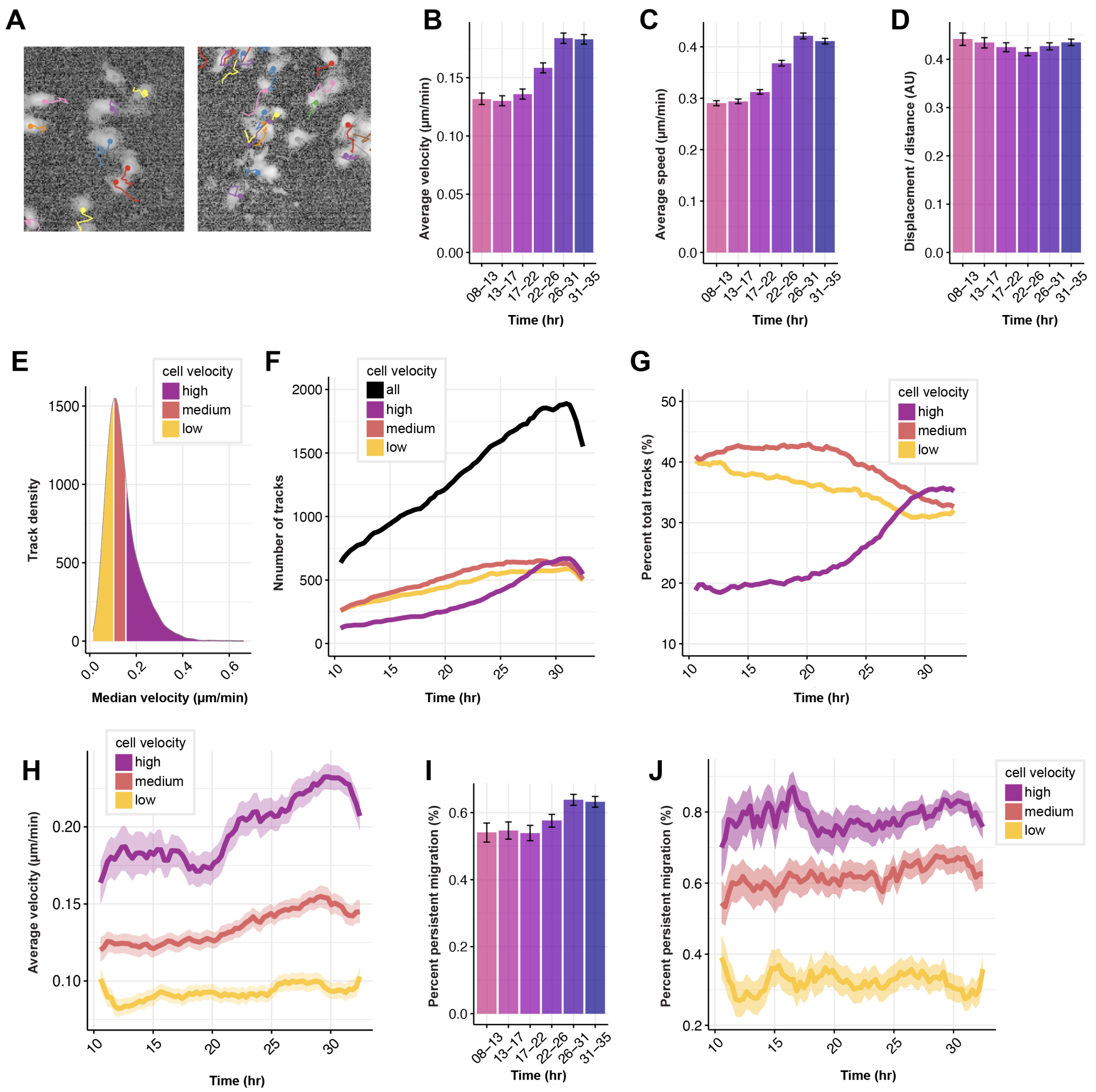

Fig. S9. Single-cell tracking of optoWnt cells in optoWnt/WT co-cultures shows increased cell migration and no change in cell persistence

a) Representative images of single-cell trajectories of mVenus-NLS+ optoWnt cells. Track color distinguishes different cells in field of view. **b)** Average cell velocities at indicated time intervals after onset of light stimulation. Graph shows mean (>1,000 tracks over 5 fields of view) ± 95% confidence interval. **c)** Average cell speed at indicated time intervals after onset of light stimulation. Graph shows mean (>1,000 tracks over 5 fields of view) ± 95% confidence interval. **d)** Average ratio of cell displacement over distance at indicated time intervals after onset of light stimulation. Graph shows mean (>1,000 tracks over 5 fields of view) ± 95% confidence interval. **e)** Distribution of median cell velocities across all cell tracks. Tracks were binned into three equal groups by median velocity (low: 0-0.15 μm/min; medium: 0.15-0.18 μm/min; high: 0.18-0.6 μm/min) as indicated. **f)** Number of tracks in each median velocity bin over time. **g)** Tracks in each velocity bin over time, shown as percentage of total tracks. **h)** Average cell velocity over time, binned by median cell velocity (low, medium, high). Graph shows mean ± 95% confidence interval. **i)** Average percent of cells undergoing persistent migration at indicated time intervals. Graph shows mean percentage ± 95% confidence interval. **j)** Average percent of cells undergoing persistent migration over time, binned by median cell velocity. Graph shows mean percentage ± 95% confidence interval.

Development • Supplementary information

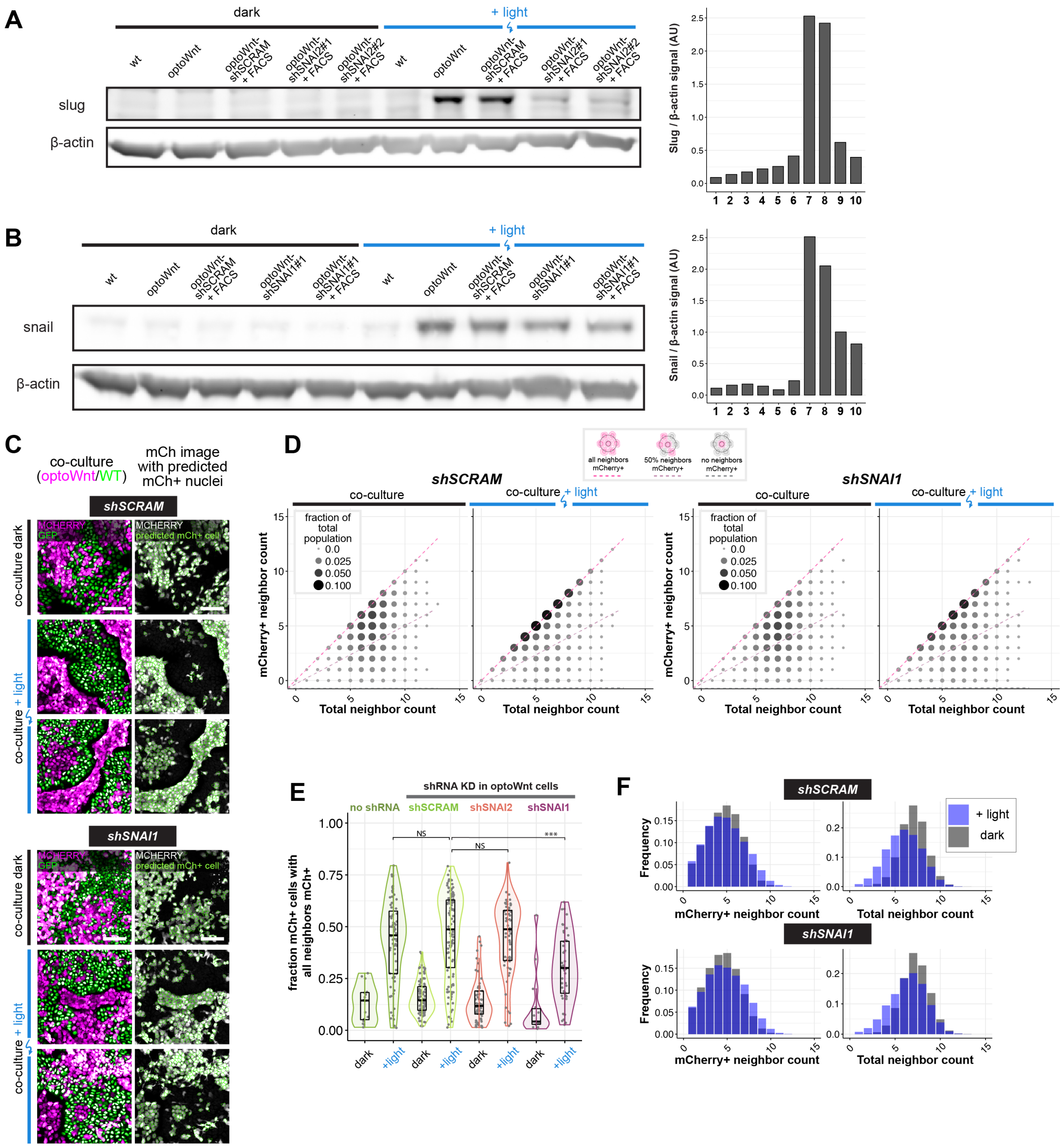

Fig. S10. Gene knockdown of EMT regulator SNAI1 in optoWnt cells shows decreased cell self-organization in co-cultures

a) Western blot (WB) image (left) for SLUG protein levels in indicated WT and optoWnt cell lines in response to shRNA knockdown. Cell lines labelled +FACS designates that lines were sorted for GFP+ expression, which marks cells infected with shRNA construct. WB quantification (right), normalized to β-actin loading control. The optoWnt line expressing shRNA snai2 #2 (+FACS) was used for subsequent experiments. **b)** Western blot image (left) for SNAIL protein levels in indicated WT and optoWnt cell lines in response to shRNA knockdown. WB quantification (right), normalized to β-actin loading control. shRNA snai1 #1 (+FACS) was used for subsequent experiments. **c)** Representative images of optoWnt/WT co-cultures (left column) in the dark and after 48 hrs illumination. OptoWnt cells express scrambled shRNA (shSCRAM, top

Development • Supplementary information

panel) or *SNAI1* shRNA (shSNAI1, bottom panel). mCherry fluorescence marks optoWnt cells, while GFP nuclear fluorescence marks WT cells. Sample images from cell neighbor analysis in CellProfiler (right column) show nuclear outline of mCh+ cells (green) overlaid on mCh channel image. Scale bar 100 μm. **d)** Cell neighbor analysis of optoWnt (mCh+) cells in optoWnt-shSCRAM/WT or optoWnt-shSNAI1/WT co-cultures kept the dark or illuminated for 48 hrs. Graph shows the count of total cell neighbors vs. count of mCh+ cell neighbors across total population of analyzed mCh+ cells (72,338 cells analyzed, pooled analysis from n=3 biological replicates). Area and color of points is proportional to the fraction of total population. Constant ratios of mCh+ to total neighbors are highlighted with dotted lines as indicated. **e)** Quantification of fraction of optoWnt (mCh+) cells whose neighbors are all mCh+ in optoWnt-shSCRAM/WT and optoWnt-shSNAI1/WT co-cultures. Each point represents an analyzed field of view (72 fields of view analyzed, n=3 biological replicates). Unpaired two-samples Wilcoxon test (p = 0.0033). **f)** Histogram of total cell neighbor counts (right) and mCh+ cell neighbor counts (left) across all analyzed cells show comparable cell densities between light and dark conditions, as well as between optoWnt-SCRAM/WT and optoWnt-SNAI1/WT co-cultures.

Development • Supplementary information

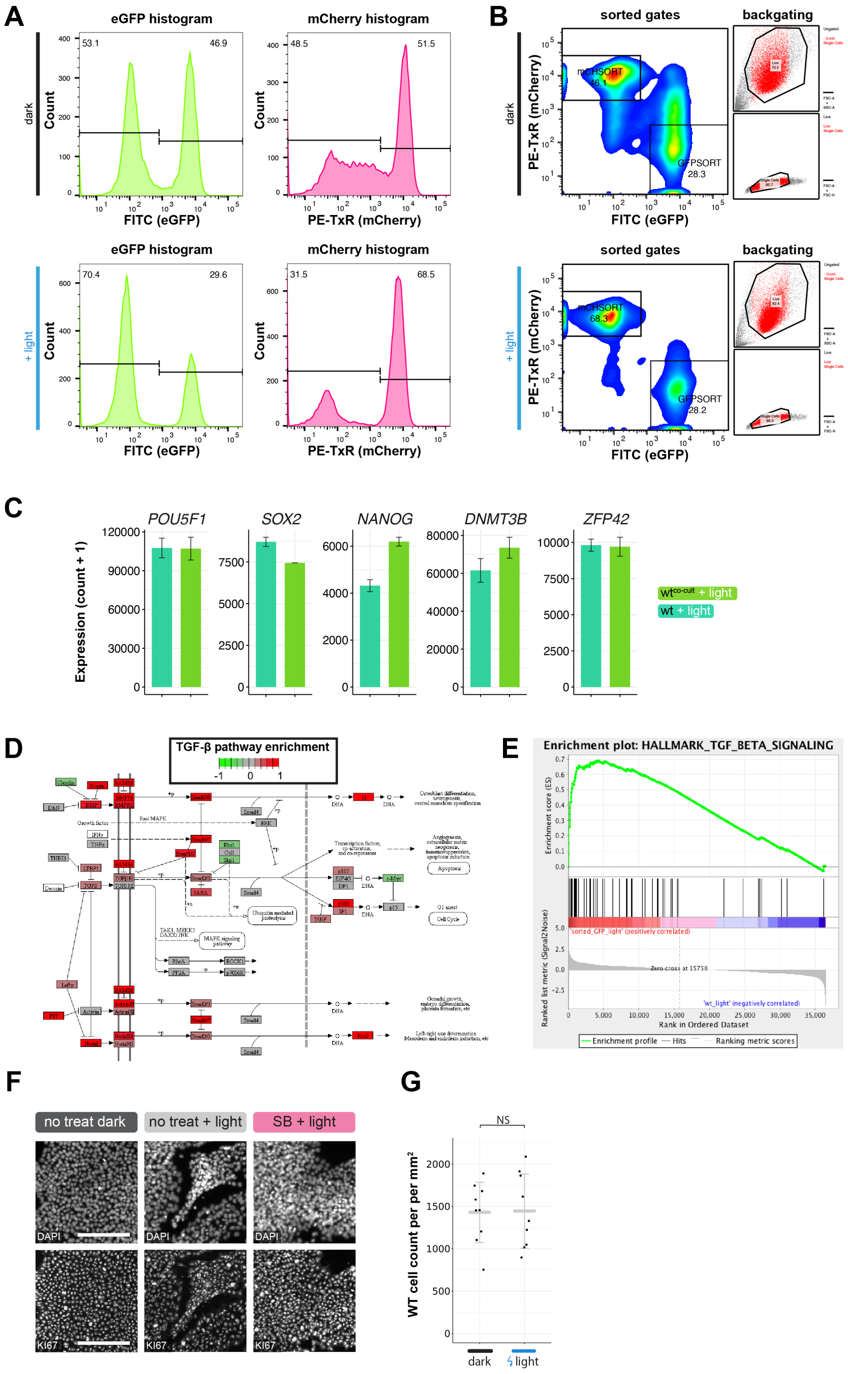

Fig. S11. RNA-seq analysis of signaling feedback between optoWnt and WT populations in co-cultures

a) Sample histograms of flow cytometry of co-cultures kept in the dark (top row) or after 48 hrs of illumination (bottom row) showing separation of the two populations: WT hESCs (GFP+) and optoWnt hESCs (mCherry+). **b)** Gating strategy for sorted cells collected for RNA seq analysis. **c)** RNA-seq results of indicated proliferation markers in WT (monoculture, illuminated) and WT (co-culture, illuminated) cells. Graphs show mean expression (read count + 1) ± 1 s.d., n = 3 biological replicates. **d)** KEGG enrichment analysis for TGF-β signaling pathway components in WT$^{\text{cocult + light}}$ over WT^{light} (monoculture) cells. Each

Development • Supplementary information

gene of TGF-β pathway is color-coded by its enrichment score, n = 3 biological replicates. **e)** Gene set enrichment analysis (GSEA) using the TGF-β hallmark gene set in $WT^{cocult + light}$ over WT^{light} (monoculture) cells, n = 3 biological replicates. **f)** Representative immunofluorescence images of optoWnt/WT co-cultures and that are untreated or under SB431542 inhibitor treatment and stained for proliferation marker Ki67. Scale bar 50 μm. **g)** Cell count of WT cells in optoWnt/WT co-cultures normalized per unit area. WT cells are defined as mCherry-negative and cell count is quantified from fluorescence imaging data. Each point represents a field of view, and graph shows mean ± 1 s.d. (one-way ANOVA, p = 0.941).

Table S1. Summary of optoWnt hESC RNA sequencing data. Sample IDs, sequencing depth, and library quality control metrics of RNA-seq study.

Click here to download Table S1

Table S2. Primary antibodies used for immunostaining and western blot (WB)

Antigen	Species	Antibody	Concentration
**LRP6**	Rbt	Ab134146	1:1000
**BRA**	Gt	RD systems AF2085	1:500
**BRA**	Rbt	Sc20109	1:500
**β-CATENIN**	Rbt	Cell sig 8480	1:500
**SLUG**	Rbt	Cell sig 9585	1:2000 (WB)
**SNAIL**	Ms	Cell sig 3895	1:1000 (WB)
**β-ACTIN**	Ms	Sigma A1978	1:5000 (WB)
**E-CAD**	Gt	RD systems AF748	1:500
**N-CAD**	Rbt	Cell sig 13116	1:500
**OCT4**	Ms	Sc5279	1:500
**SOX2**	Rbt	Ab97959	1:500
**NANOG**	Ms	Ab62734	1:500
**KI67**	Ms	Cell sig 9449	1:200
**pSMAD1/5**	Rbt	Cell sig 9516	1:200
**pSMAD2/3**	Rbt	Cell sig 18338	1:200
**MCHERRY**	Ck	Ab205402	1:1000
**MVENUS**	Rbt	Ab6556	1:1000

Development • Supplementary information

Table S3. qPCR primers and gene knockdown shRNAs

shRNA	Sequence Forward (5’→3’)
shSNAI2 #1 (TRCN0000015389)	CCGGCCCATTCTGATGTAAAGAAATCTCGAGATTTCTTTACATCAGAATGGGTTTTTG
shSNAI2 #2 (TRCN0000284362)	CCGGGAGTGACGCAATCAATGTTTACTCGAGTAAACATTGATTGCGTCACTCTTTTTG
shSNAI1 (TRCN0000063818)	CCGGCCACTCAGATGTCAAGAAGTACTCGAGTACTTCTTGACATCTGAGTGGTTTTTG
shSCRAM	CCGGCCTAAGGTTAAGTCGCCCTCGCTCGAGCGAGGGCGACTTAACCTTAGGTTTTTG

qPCR primer	Sequence Forward (5’→3’)	Sequence Reverse (5’→3’)
POU5F1	ATGCATTCAAACTGAGGTGCCTGC	AACTTCACCTTCCCTCCAACCAGT
NANOG	CCCAAAGGCAAACAACCCACTT	AGCTGGGTGGAAGAGAACACA
SOX1	CAGTACAGCCCCATCTCCAAC	GCGGGCAAGTACATGCTGA
PAX6	TGGGCAGGTATTACGAGACTG	ACTCCCGCTTATACTGGGCTA
SOX17	GGACCGCACGGAATTTGAAC	TAATATACCGCGGAGCTGGC
OTX2	CAAAGTGAGACCTGCCAAAAAGA	TGGACAAGGGATCTGACAGTG
T	TTTCCAGATGGTGAGAGCCG	CCGATGCCTCAACTCTCCAG
EOMES	GCCATGCTTAGTGACACCGA	GGACTGGAGGTAGTACCGC
MIXL	GGCGTCAGAGTGGGAAATCC	GGCAGGCAGTTCACATCTACC
GATA6	CTCAGTTCCTACGCTTCGCAT	GTCGAGGTCAGTGAACAGCA
MSX1	ACACAAGACGAACCGTAAGCC	CACATGGGCCGTGTAGAGTC
GATA4	CGACACCCCAATCTCGATATG	GTTGCACAGATAGTGACCCGT
CDH1	CGAGAGCTACACGTTCACGG	GGGTGTCGAGGGAAAAATAGG
CDH2	TCAGGCGTCTGTAGAGGCTT	ATGCACATCCTTCGATAAGACTG
VIM	AGTCCACTGAGTACCGGAGAC	CATTTCACGCATCTGGCGTTC
SNAI1	TCGGAAGCCTAACTACAGCGA	AGATGAGCATTGGCAGCGAG
SNAI2	CGAACTGGACACACATACAGTG	CTGAGGATCTCTGGTTGTGGT
18S (RPS18)	CTTCCACAGGAGGCCTACA	CTTCGGCCCACACCCTTAAT

Development • Supplementary information

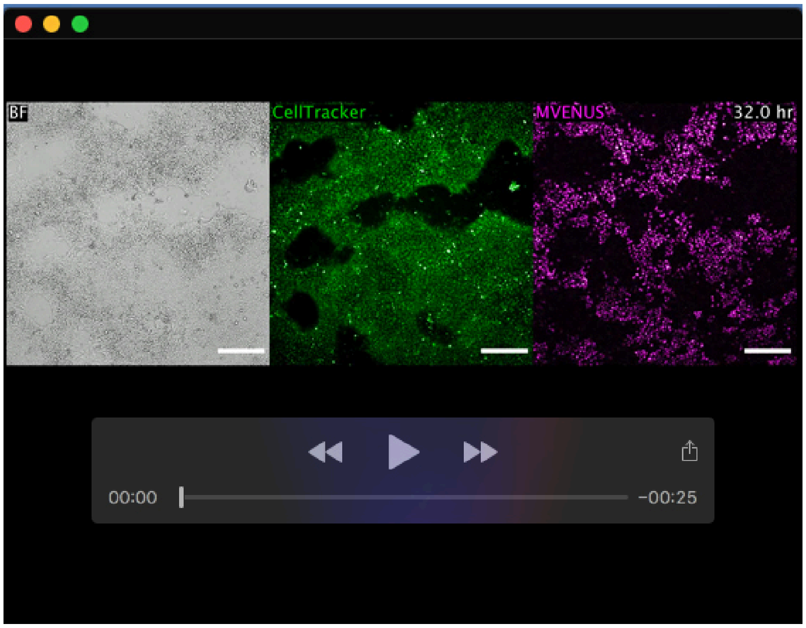

Movie 1. 2D co-culture timelapse imaging. Timelapse optoWnt/WT co-cultures under optogenetic stimulation. Three image channels displayed: brightfield (BF, grey), CellTracker membrane dye fluorescence (green), and optoWnt-mVenus-NLS cells (magenta). Time after onset of illumination indicated. Scale bar 250 μm.

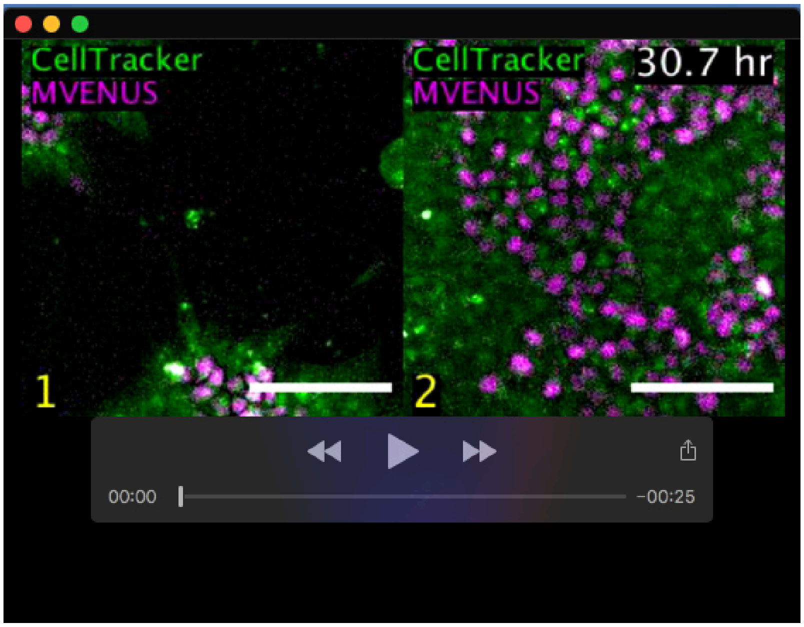

Movie 2. 2D co-culture timelapse imaging (zoom). Zoom-in of two fields of view of Supplementary Video 2. Left: cell aggregation occurs outside hESC colony ; right: cell aggregation occurs inside hESC colony. Merge of CellTracker membrane dye fluorescence (green) and optoWnt-mVenus-NLS cells (magenta). Time after onset of illumination indicated. Scale bar 100 μm.

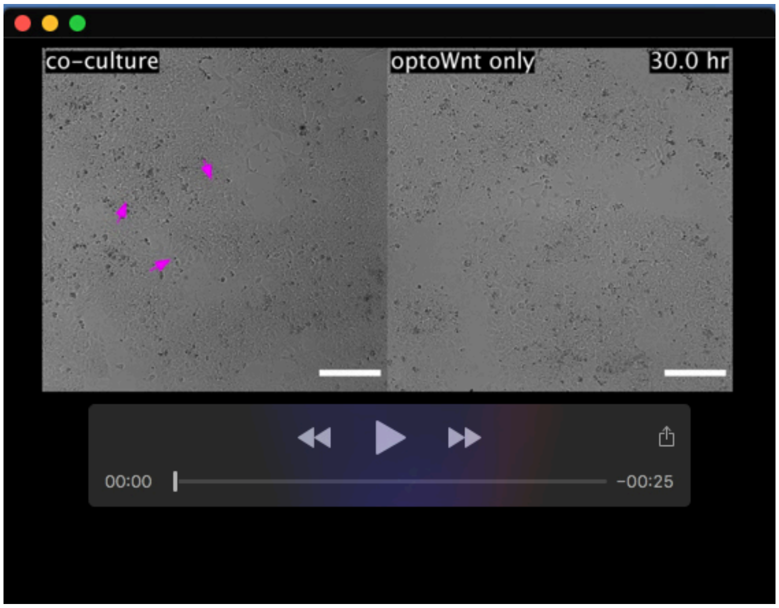

Movie 3. Aggregate dynamics in 2D co-cultures vs. monocultures. Brightfield timelapse optoWnt/WT co-culture (left) and optoWnt monoculture (right) under optogenetic stimulation. Magenta arrows highlight aggregates that display collective movement and aggregate fusion. Time after onset of illumination indicated. Scale bar 250 μm.

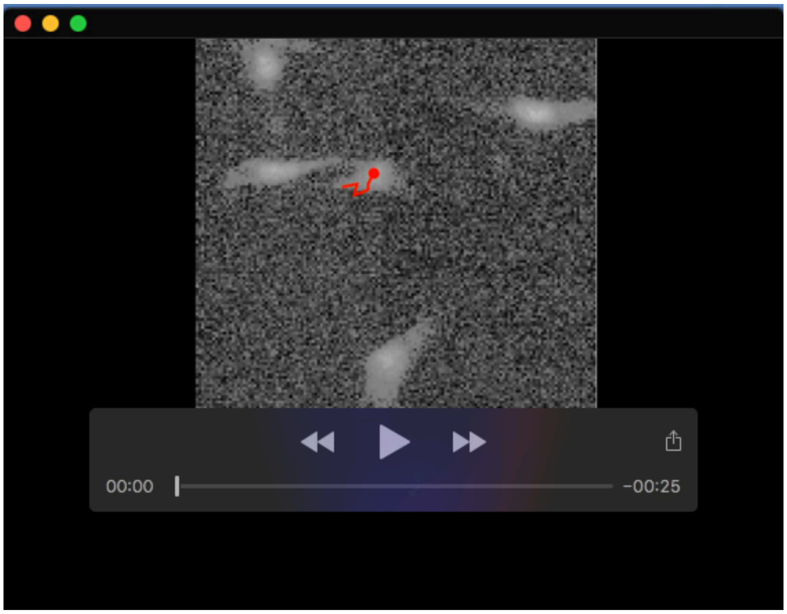

Movie 4. Sample single-cell track of optoWnt-mVenus-NLS cell under optogenetic stimulation in optoWnt/WT co-culture. Timelapse of optoWnt/WT co-culture under optogenetic stimulation, mVenus channel.

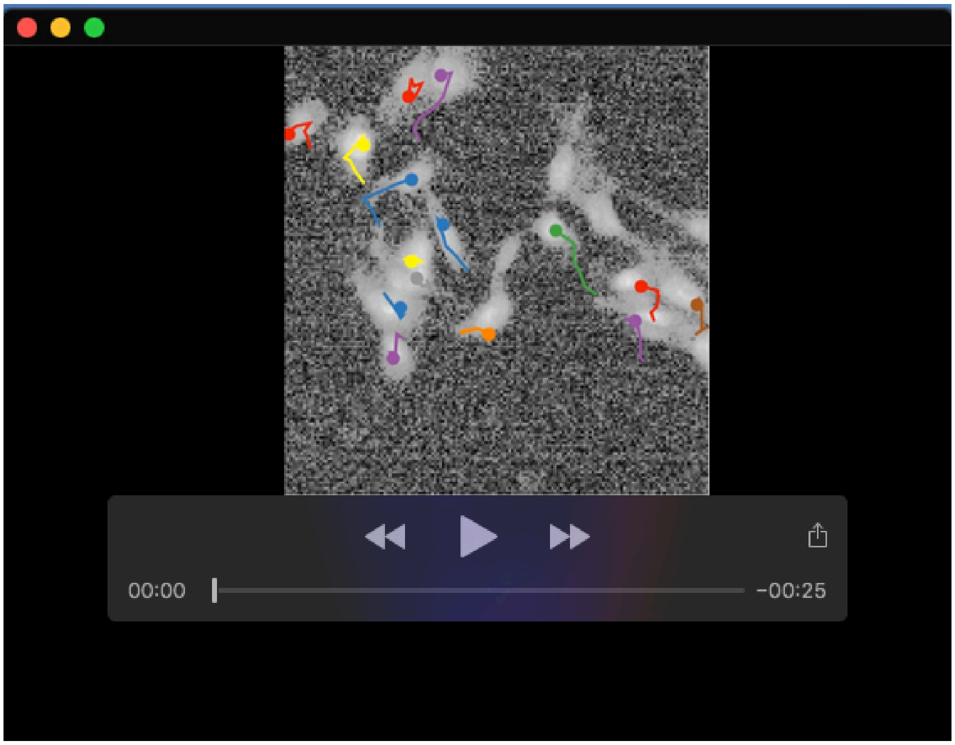

Movie 5. Multiple single-cell tracks of optoWnt-mVenus-NLS cell under optogenetic stimulation in optoWnt/WT co-culture. Timelapse of optoWnt/WT co-culture under optogenetic stimulation, mVenus channel.
