# Supplementary material for: Optogenetic control of Wnt signaling models cell-intrinsic embryogenic patterning using 2D human pluripotent stem cell culture
Source: Development. 2023 Jul 26;150(14):dev201386. doi: 10.1242/dev.201386 (PMC10399980; doi:10.1242/dev.201386)
Supplement: Supplementary file 1 [file develop-150-201386_review_history.pdf]

Optogenetic control of Wnt signaling models cell-intrinsic embryogenic patterning using 2D human pluripotent stem cell culture

Nicole A. Repina, Hunter J. Johnson, Xiaoping Bao, Joshua A. Zimmermann, David A. Joy, Shirley Z. Bi, Ravi S. Kane and David V. Schaffer
DOI: 10.1242/dev.201386

Editor: Matthias Lutolf

Review timeline

Original submission:	18 October 2022
Editorial decision:	24 November 2022
First revision received:	22 May 2023
Editorial decision:	15 June 2023
Second revision received:	21 June 2023
Accepted:	21 June 2023

Original submission

First decision letter

MS ID#: DEVELOP/2022/201386

MS TITLE: Optogenetic control of Wnt signaling models cell-intrinsic embryogenic patterning using 2D human pluripotent stem cell culture

AUTHORS: Nicole A Repina, Hunter J Johnson, Xiaoping Bao, Joshua A Zimmermann, David A Joy, Ravi S Kane, and David V Schaffer

I have now received all the referees' reports on the above manuscript, and have reached a decision. The referees' comments are appended below, or you can access them online: please go to BenchPress and click on the 'Manuscripts with Decisions' queue in the Author Area.

As you will see, the referees express considerable interest in your work, but have some significant criticisms and recommend a substantial revision of your manuscript before we can consider publication. If you are able to revise the manuscript along the lines suggested, which may involve further experiments, I will be happy receive a revised version of the manuscript. Your revised paper will be re-reviewed by one or more of the original referees, and acceptance of your manuscript will depend on your addressing satisfactorily the reviewers' major concerns. Please also note that Development will normally permit only one round of major revision. If it would be helpful, you are welcome to contact us to discuss your revision in greater detail. Please send us a point-by-point response indicating your plans for addressing the referee's comments, and we will look over this and provide further guidance.

Please attend to all of the reviewers' comments and ensure that you clearly highlight all changes made in the revised manuscript. Please avoid using 'Tracked changes' in Word files as these are lost in PDF conversion. I should be grateful if you would also provide a point-by-point response detailing how you have dealt with the points raised by the reviewers in the 'Response to Reviewers' box. If you do not agree with any of their criticisms or suggestions please explain clearly why this is so.

© 2023. Published by The Company of Biologists under the terms of the Creative Commons Attribution License (https://creativecommons.org/licenses/by/4.0/).

Reviewer 1

Advance summary and potential significance to field

In this manuscript, the authors use an optogenetic system to control the activation of Wnt signaling intracellular cascade (optoWnt). They use this system to study how the cell-intrinsic variability of Wnt activity in a human Pluripotent Stem Cells (hPSC) population contributes to self-organization reminiscent of primitive streak behavior during gastrulation.

The authors showed that:
1) Activation of Wnt signaling pushes hPSC towards a primitive streak (PS)/mesendodermal cell fate and EMT as shown in many publications.
2) Upon activation of Wnt pathway cell sorting occurs: Wnt-induced cells set apart from the wild-type cells (non Wnt-induced).

3) The sorting of Wnt-active PS cells seems to be due to the activation of an EMT program and to increased cell motility. The authors show that the cell sorting mechanism is dependent on TGFb activity downstream of Wnt pathway activation.
Of note, differential cell proliferation does not seem to play a role in cell sorting.

4) It seems that the secretion of BMP ligands from the Wnt-induced cells triggers a BMP response in the wild-type (non Wnt-induced) cells. The authors suggest that BMP signals from the PS cells could contribute to a cell priming effect of epiblast cells for subsequent cell specification.

In my opinion, the manuscript is well written and most of the experiments are very well controlled. OptoWnt is a useful tool for dissecting intercellular mechanisms that contribute to human early development. I would have liked to see the spatial control aspect of the optoWnt system explored more, but I also understand that it could be part of a following manuscript. In summary, I find that the manuscript fits the interest of this scientific journal and it is definitely worth of consideration.

Comments for the author

Below I list some points that I would like the authors to address:
1) The authors concluded that increased cell motility in PS (optoWnt light-activated) cells is responsible for the cell sorting behavior observed in their setup.
The increased cell motility can explain why the PS cells move away from epithelial wild-type (non Wnt-induced) cells. Although, it does not explain in my opinion why cells form stable PS "aggregates". What keeps PS cells together when they find each other? I think differential cell-cell adhesion (eventually driven by different cadherin classes) can have a role in this process, as already reported in other contexts. I would like the authors to comment on this point.
2) Experiments with treatments that affect cell motility (e.g. blebbistatin, and maybe Y27632) could reinforce the claim that self-organization of optoWnt and wild-type cells is mainly dependent on increased cell motility.
3) The authors showed that treatment with a TGFb inhbitor (SB431542) antagonizes EMT and increased cell motility resulting in the loss of cell sorting behavior.
TGFb signaling is known to be required for primitive streak/mesendodermal differentiation. Is the self-organization impacted because TFGb inhibition affects EMT and cell motility or because TGFb inhibition precludes the differentiation of hPSC towards PS cells? What is the impact of SB treatment on the number of BRA+ cells?
4) The authors showed that activation of Wnt signaling in optoWnt cells causes an increase in BMP pathway activity in these cells. The increase in BMP activity seems to be even more pronounced than what they record for TGFb pathway. Does BMP inhibition have an effect on the self-organization of the system?
5) The authors claim that the cell sorting behavior observed in their system is not due to differential proliferation rate of activated optWnt cells.
Indeed, they show that optoWnt cells do not change substantially their rate of proliferation once activated.
Could it be that the activation of the optoWnt cells results in changes of proliferation rate of the wild-type cells? I suggest the author to check the EdU staining in a time-course experiment for the wild-type cells co-cultured with activated optoWnt cells.

© 2023. Published by The Company of Biologists under the terms of the Creative Commons Attribution License (https://creativecommons.org/licenses/by/4.0/).

6) The authors claim that the increased BMP activity seen in wild-type cells, when co-cultured with activated optoWnt cells, is probably due to secretion of diffusible signals (very likely BMP ligands). To reinforce their claim the authors could check if they phenocopy the effect on wild-type cells exposing them to activated optWnt cells-conditioned medium. Moreover, they could check the effect of BMP ligands inhibitors (e.g. Noggin) on wild-type cells in the co-culture setup.
7) BMP ligands have been shown in some hPSC differentiation contexts to induce a primitive streak/mesendodermal fate (for example in Warmflash A. et al Nat. Methods, 2014, DOI: 10.1038/nmeth.3016 to cite just one example). In their setup, the authors see the induction of BMP pathway in wild-type cells without conversion to a mesendodermal fate. Is the difference in cell fate specification linked to different levels of BMP stimulation? Alternatively, are the optoWnt activated cells precluding the differentiation of the wild-type cells via other mechanisms? I would like the author to comment on this point.

Reviewer 2

Advance summary and potential significance to field

In this study, Repina and coworkers study the consequences of light-induced Wnt signaling in human embryonic stem cells (hESCs). Both the optoWnt optogenetic tool and the engineered optoWnt hESC line used in this study were previously published, but prior studies have not used these optogenetics tools to examine the question of how cells with different levels of Wnt activity might self-organize into distinct domains. Broadly, the authors find that a heterogeneous mixture of optoWnt and WT cells segregates into distinct domains, presumably through an EMT-like process and cell sorting by differential adhesion. I have been aware of this interesting work for some time since it became available as a preprint and am very happy to see it considered for publication at Development. The paper is well-written, the data solid, and the findings of broad interest to a developmental audience.

Comments for the author

I would like to raise a few issues regarding the authors' conceptual claims.
(1) One major conceptual objection involves the authors' invoking of "Turing-like" patterns in the discussion, which suggests a reaction-diffusion system (e.g. diffusible activators and inhibitors that act on cells and regulate their own secretion) to spontaneously form patterns. This is misleading in at least two ways:
- The authors spend most of the manuscript arguing that the pattern emerges through a cell sorting/cell migration mechanism, entirely different than patterning via reaction-diffusion on a fixed field of cells.
- The experimental system does not involve stochastic symmetry breaking in the classic sense of what "Turing-like" patterns purport to explain. In a Turing pattern, small fluctuations in an otherwise uniform field of cells are amplified by feedback to form bifurcated states and spatial patterns. Here, the inhomogeneity is "baked in" directly by the 1:1 co-culture and light stimulation.
- Perhaps very dim or transient illumination, leading to beta-catenin levels that are only very slightly above wild-type, would also self-amplify into distinct domains. This scenario would be more plausibly explained by Turing-like feedback to amplify small asymmetries into large spatial domains. The authors do not need to carry out this experiment, but it would lend weight to a Turing-like model if that is a claim they feel strongly about making.
Based on these arguments, I feel that it would be worth pointing out in the Discussion that these results are not "Turing-like" in themselves (either in mechanism or conceptual framework). At the very least, it should clearly consider these points and refute them, rather than make the unsupported claim.

(2) I am not fully convinced by the authors' claim that Nodal/TGFbeta signaling is essential for symmetry breaking. I don't want to suggest that the authors have to answer every question below, but at least some picture of what is going on (and how Nodal is involved) would be helpful:
- Figure 4I is the strongest data that Nodal signaling is important, but is hard to interpret. Although the clumps of opto-Wnt cells are smaller, sorting into distinct domains is still evident!

© 2023. Published by The Company of Biologists under the terms of the Creative Commons Attribution License (https://creativecommons.org/licenses/by/4.0/).

- How is Nodal's effect supposedly mediated? Does sorting initiate similarly but arrest earlier in Nodal-inhibited cells? Does Nodal inhibition affect opto-Wnt cells, wild-type cells, or both? Could the results of 4J-K simply be explained by some baseline cell death due to nodal inhibition decreasing proliferation, or a change in overall cell motility but not the sorting program?
- Do Nodal-inhibited OptoWnt cells still show changes in Ecad/Snail expression? (If so, this would suggest a mechanism for its action. If not, it is very hard to understand what role it might play to specifically regulate cell sorting.)
- Is Smad phosphorylation equally induced in both wild-type and optoWnt cells to equal extents during co-culture? The authors only quantify the effect in wild-type cells, not optoWnt cells, in Figure 5F-G. However, the inset images indicate uniform Smad activation in both cell populations, suggesting that Nodal signaling is uniformly induced in all cells. The authors should quantify Nodal in the two cell populations during co-culture as in Figure 5F-G.
- Figure 5G may show a statistically significant effect, but its magnitude is so small (~0.18 AU vs ~0.2 AU) that it is hard to interpret as biologically meaningful.

(3) The authors claim that their data suggest "that Wnt alone is sufficient for initiating the human BMP4-Wnt-Nodal primitive streak feedback loop without the need for exogenous BMP4 stimulation". I cannot say for sure whether this has ever been reported in hESCs, but certainly this well established in mouse ESCs, for instance in Martinez-Arias' early gastruloid papers, which robustly develop A-P axes in response to CHIR treatment without BMP but are not cited in the Discussion section related to this point.

(4) The authors discuss EMT at length, but this might invite considerable controversy from the ES cell field, as many authors are careful to avoid suggesting a true "epithelial-to-mesenchymal" transition because embryonic stem cells do not form a true epithelium, and instead refer to an EsMT (embryonic stem cell to mesenchymal transition); see e.g. Cermola et al, Stem Cell Rep 2022. It might be prudent to amend the terminology used for this transition.

Otherwise, I am very much in favor of publication and congratulate the authors on a fine piece of work.

First revision

<u>Author response to reviewers' comments</u>

<u>Reviewer 1)</u>

1)The authors concluded that increased cell motility in PS (optoWnt light-activated) cells is responsible for the cell sorting behavior observed in their setup.
The increased cell motility can explain why the PS cells move away from epithelial wild-type (non Wnt-induced) cells. Although, it does not explain in my opinion why cells form stable PS "aggregates". What keeps PS cells together when they find each other? I think differential cell-cell adhesion (eventually driven by different cadherin classes) can have a role in this process, as already reported in other contexts. I would like the authors to comment on this point.

1. Thank you for this comment. In line with the hypothesis that differential cell-cell adhesion may be playing a role in self-adhesion or repulsion of the two populations, we had previously tested knock-downs of cadherins such as E- and N-cadherin in the optoWnt and/or WT populations. However, we did not see a clear phenotype or negative effect on segregation. From the RNA-seq results, we found that multiple cadherins and cell adhesion proteins are differentially expressed upon optoWnt-induced differentiation (see figure below). Therefore, we concluded that segregation based on differential adhesion is likely due to a combinatorial effect of cell adhesion proteins that is subject for a follow-up manuscript. We also added a comment in the Discussion section on the potential role of differential cell-cell adhesion, also reproduced below:

"The incomplete reduction of self-segregation under cell migration inhibitor treatment (Figure 4G-I) suggests that while cell motility is required for large-scale aggregation, motility works in combination with other cell state changes associated with differentiation and EMT, such as for example differential cell-cell adhesion (Steinberg and Takeichi, 1994), to mediate pattern shape

© 2023. Published by The Company of Biologists under the terms of the Creative Commons Attribution License (https://creativecommons.org/licenses/by/4.0/).

and stability."

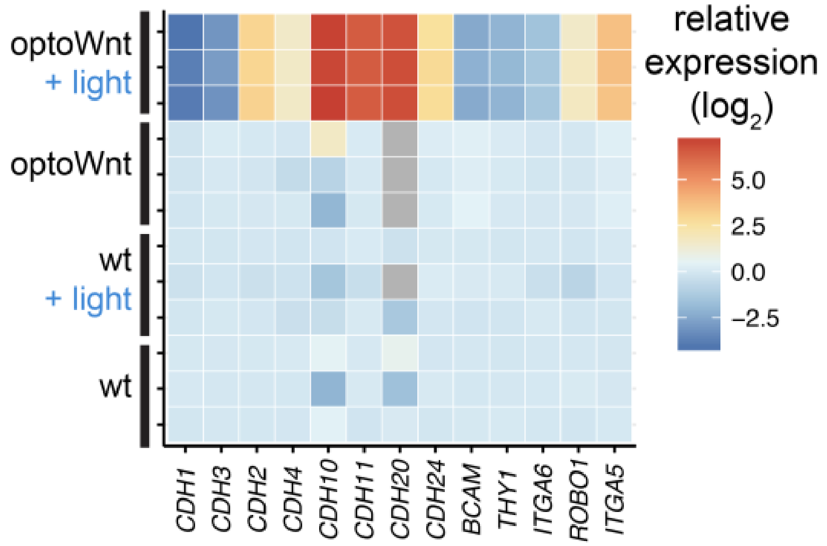

2) Experiments with treatments that affect cell motility (e.g. blebbistatin, and maybe Y27632) could reinforce the claim that self-organization of optoWnt and wild-type cells is mainly dependent on increased cell motility.

2. Thank you for this helpful suggestion. We have performed the suggested perturbations of co-cultures with the cell motility inhibitors blebbistatin and ROCK inhibitor Y-27632, quantified the effect on cell segregation relative to untreated co-cultures, and added them as main Figures 4G, 4H, and 4I of the manuscript (shown below).

In summary, we found blebbistatin to be toxic to hESCs even at low doses (5 µM), whereas ROCK inhibitor Y-27632 led to an altered phenotype of optoWnt aggregates (Figure 4G-H). Though optoWnt and WT cells still segregated, treatment with Y-27643 resulted in smaller, more abundant, and more circular optoWnt aggregates that did not display the apparent contact area minimization between the optoWnt and WT domains (Figure 4I). This effect is consistent with optoWnt cell aggregation due to local cell-cell interactions without larger- scale cell migration out of and within epithelial hESC colonies. These results suggest that cell migration is required for larger aggregate size and their distinct spatial separation, and that cell migration works in combination with cell state changes during differentiation and EMT to mediate segregation.

Figure 4

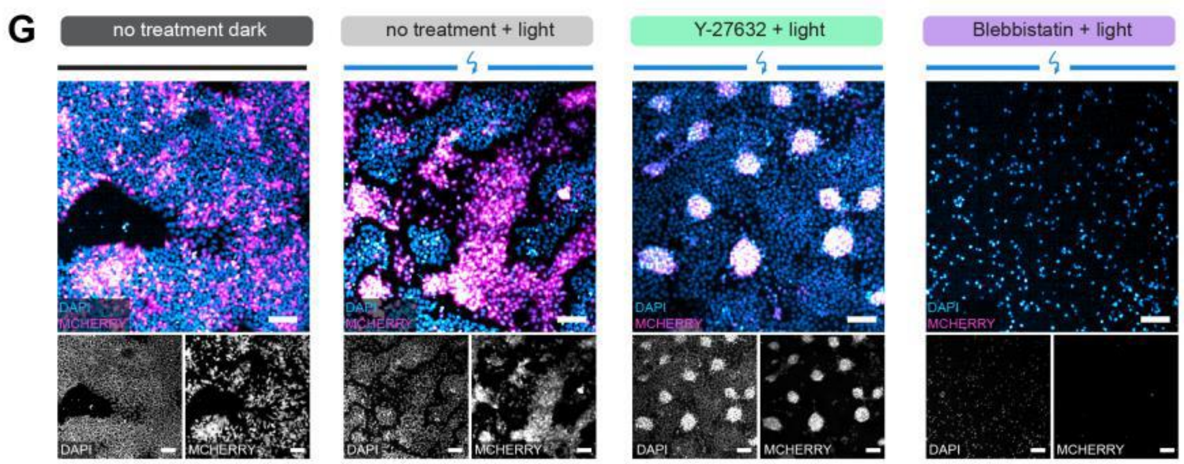

© 2023. Published by The Company of Biologists under the terms of the Creative Commons Attribution License (https://creativecommons.org/licenses/by/4.0/).

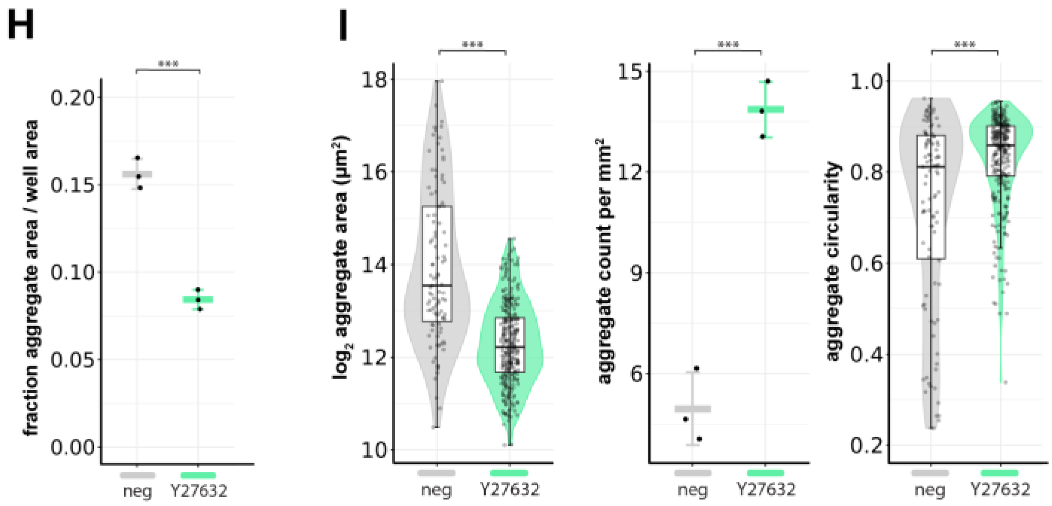

3)The authors showed that treatment with a TGFb inhbitor (SB431542) antagonizes EMT and increased cell motility resulting in the loss of cell sorting behavior.
TGFb signaling is known to be required for primitive streak/mesendodermal differentiation. Is the self-organization impacted because TFGb inhibition affects EMT and cell motility or because TGFb inhibition precludes the differentiation of hPSC towards PS cells? What is the impact of SB treatment on the number of BRA+ cells?

3. Thank you for this comment. To distinguish the contribution of TGF-□ signaling to cell motility/EMT vs. differentiation, we performed new co-culture experiments in the presence or absence of TGF-□ inhibitor (SB431542) and quantified the effect on differentiation markers (e.g. BRA) and EMT markers (e.g. ECAD and NCAD). The results of these experiments were added as main Figure 5G, 5H, and supplementary figure S5B.

Interestingly, we found that though SB treatment inhibited cell segregation, optoWnt cells in these cultures still expressed BRA and underwent the ECAD to NCAD switch, suggesting they were able to undergo initial stages of differentiation and EMT (Figure 5G-J). However, though expression of ECAD decreased under SB treatment, there was still detectable expression suggesting an incomplete EMT (Figure 5J). SB treatment also did not show an observable effect on cell proliferation, as measured with Ki67 staining (Supplementary Figure S11F). These results suggest that TGF-□ signaling is necessary for optoWnt cells to fully undergo EMT.

Figure 5

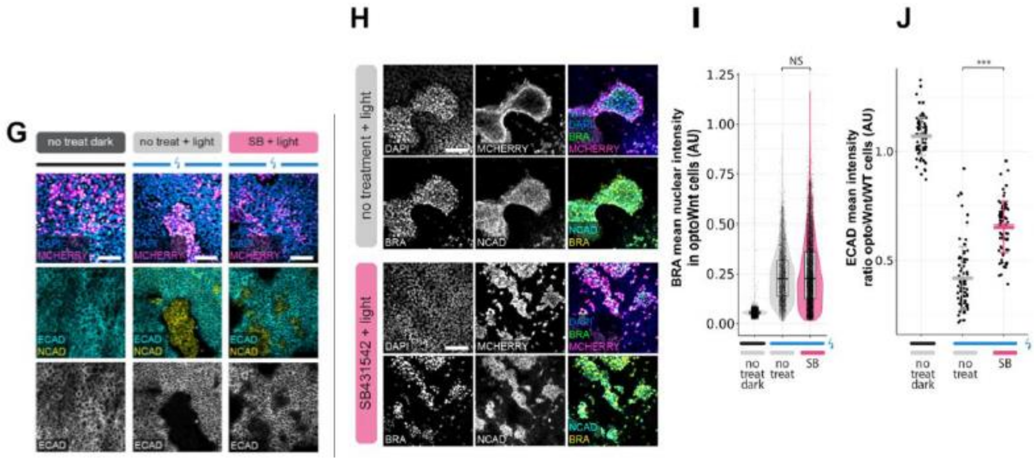

Supplementary Figure 11

© 2023. Published by The Company of Biologists under the terms of the Creative Commons Attribution License (https://creativecommons.org/licenses/by/4.0/).

F

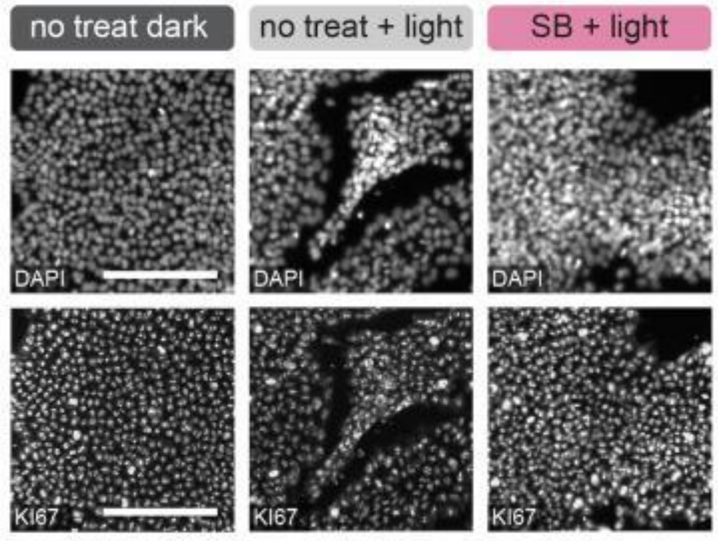

4)The authors showed that activation of Wnt signaling in optoWnt cells causes an increase in BMP pathway activity in these cells. The increase in BMP activity seems to be even more pronounced than what they record for TGFb pathway. Does BMP inhibition have an effect on the self-organization of the system?

4. Thank you for this comment. To address whether BMP inhibition has an effect on cell segregation, we have now performed co-culture experiments in the presence of the BMP pathway inhibitors Noggin and LDN-193189 and quantified the effect on aggregate size and cell segregation. In this screen we also included perturbation with IWP-2 (inhibitor of Wnt secretion) and SB431542. The results of this experiment are shown in main Figures 5B, 5C, 5D. We found that BMP inhibition had no detectable effect on self-organization of the system or aggregate morphology.

Figure 5

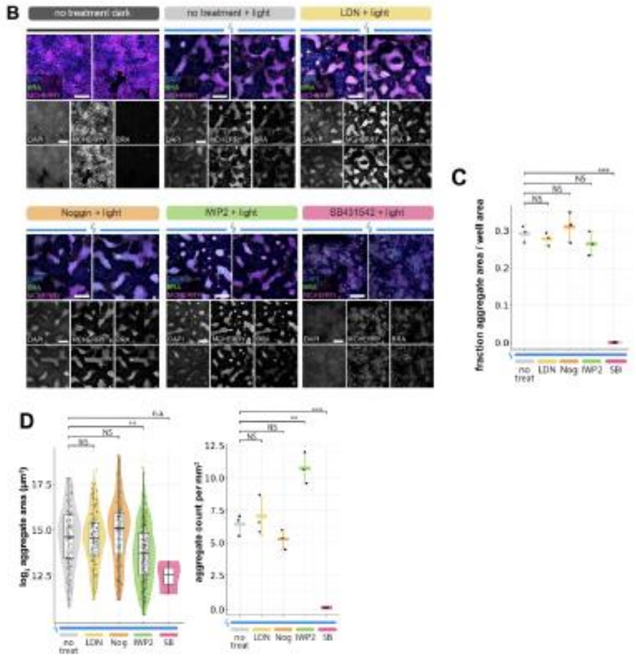

© 2023. Published by The Company of Biologists under the terms of the Creative Commons Attribution License (https://creativecommons.org/licenses/by/4.0/).

5)The authors claim that the cell sorting behavior observed in their system is not due to differential proliferation rate of activated optWnt cells. Indeed, they show that optoWnt cells do not change substantially their rate of proliferation once activated. Could it be that the activation of the optoWnt cells results in changes of proliferation rate of the wild-type cells? I suggest the author to check the EdU staining in a time-course experiment for the wild-type cells co-cultured with activated optoWnt cells.

5. We appreciate the question. The rate of proliferation of optoWnt cells increased slightly upon light stimulation, as evidenced by the increase in EdU incorporation in Supplementary Fig. S7 D-E. The increase in proportion of optoWnt : WT cells in co-culture is also evidenced in Fig S11A, where in the dark the ratio is ~ 50 : 50 %, whereas after 48 hrs illumination it is ~ 70 : 30 %. This shows that activated optoWnt cells have a higher proliferation rate upon activation. However, the higher abundance of optoWnt cells does not seem to impact cell segregation. Specifically, if we decrease the relative number of optoWnt cells in culture, segregation still occurs (Supplementary Fig S5). Furthermore, the segregation does not seem to be a result of clonal expansion of optoWnt cells (Supplementary Fig S7F). We updated the manuscript text accordingly to improve clarity.

To address the question whether activated optoWnt cells could change the proliferation rate of the WT cells, we quantified whether there is a change in cell number in the WT population in light vs. dark conditions. We detected no significant difference in WT cell counts in co- culture with optoWnt cells between dark and light conditions, as shown below. This figure is added as Supplementary Figure S11G. Furthermore, we do not observe any significant changes in proliferation as shown via Ki67 staining (Supplementary Figure S11F, please see figure in Reviewer 1 comment 3).

Supplementary Figure 11

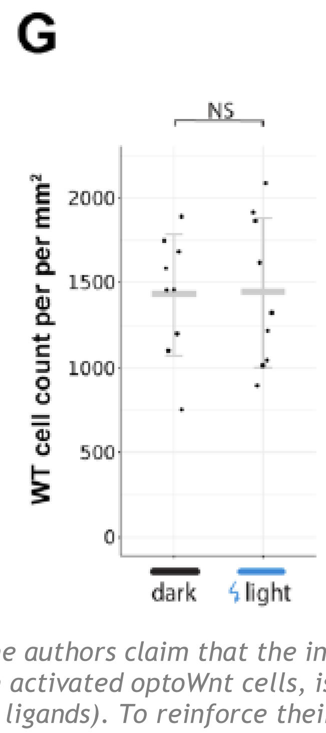

6)The authors claim that the increased BMP activity seen in wild-type cells, when co-cultured with activated optoWnt cells, is probably due to secretion of diffusible signals (very likely BMP ligands). To reinforce their claim the authors could check if they phenocopy the effect on wild-type cells exposing them to activated optWnt cells-conditioned medium. Moreover, they could check the effect of BMP ligands inhibitors (e.g. Noggin) on wild-type cells in the co-culture setup.

6. To address this comment, we performed the suggested experiment of BMP ligand inhibition during co-culture (please see inhibitor experiment results in Reviewer 1 comment 4). Further, we quantified the expression of the BMP downstream effector pSMAD1/5 with

© 2023. Published by The Company of Biologists under the terms of the Creative Commons Attribution License (https://creativecommons.org/licenses/by/4.0/).

immunostaining of co-cultures grown with or without the presence of the BMP inhibitors Noggin and LDN-193189. Indeed, upon light stimulation we observed upregulation of both pSMAD1/5 and pSMAD2/3 in WT cells. BMP ligand inhibitor treatment specifically decreased pSMAD1/5 expression, with a minor effect on pSMAD2/3, in both WT and optoWnt cells. These results support that BMP and TGF-□ is induced in WT cells via diffusible signals from optoWnt cells, and they are added as main Figure 6F, 6G, 6H, and 6I. We believe this experiment, particularly the addition of Noggin that directly binds and inhibits key BMP ligands, addresses the role of such secreted ligands in the observed behavior.

Figure 6

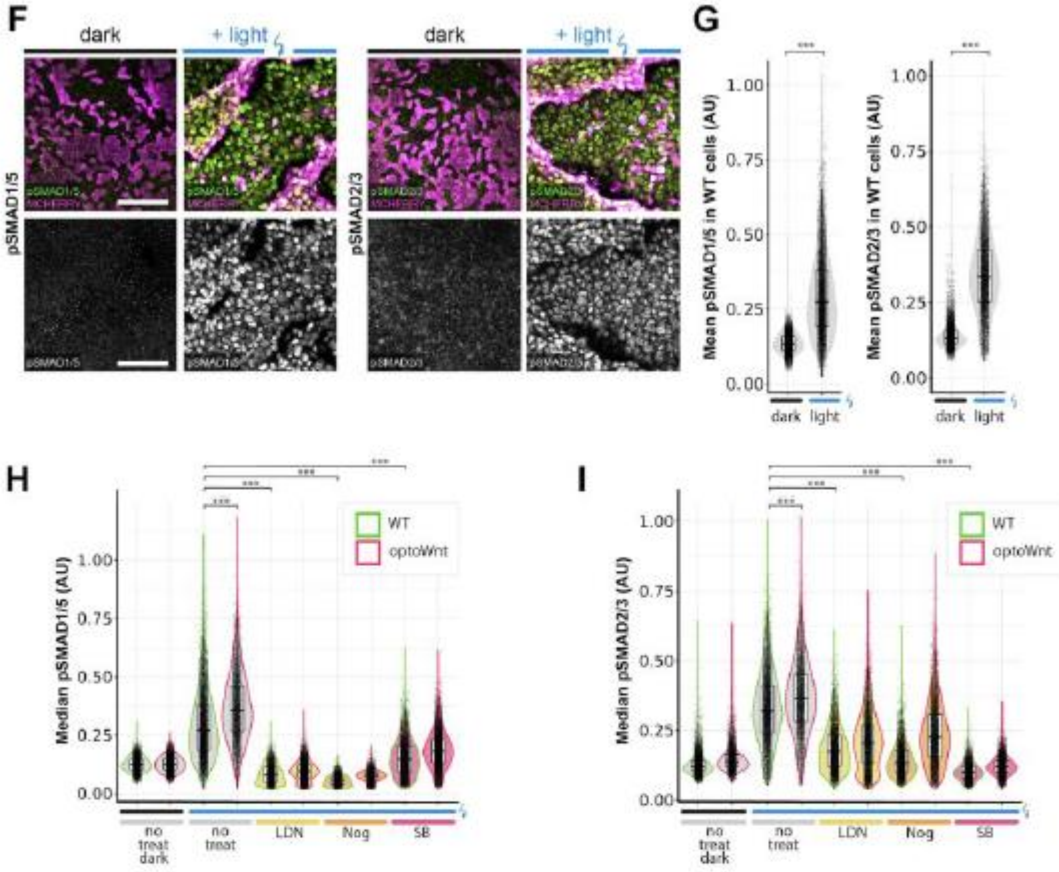

7) BMP ligands have been shown in some hPSC differentiation contexts to induce a primitive streak/mesendodermal fate (for example in Warmflash A. et al, Nat. Methods, 2014, DOI: 10.1038/nmeth.3016 to cite just one example). In their setup, the authors see the induction of BMP pathway in wild-type cells without conversion to a mesendodermal fate. Is the difference in cell fate specification linked to different levels of BMP stimulation? Alternatively, are the optoWnt activated cells precluding the differentiation of the wild-type cells via other mechanisms? I would like the author to comment on this point.

7. The reviewer brings up an interesting point, and the lack of a clear differentiation phenotype in the WT cells may due to a combination of factors. It could be that the secreted BMP ligand has a low effective concentration due to dilution in the surrounding tissue culture media volume. In addition, the hESCs are cultured in a pluripotency media, which contains pluripotency maintenance factors FGF2 and TGF-□, which may overpower the effect of secreted BMP and preclude a clear differentiation phenotype.

© 2023. Published by The Company of Biologists under the terms of the Creative Commons Attribution License (https://creativecommons.org/licenses/by/4.0/).

Reviewer 2

(1) One major conceptual objection involves the authors' invoking of "Turing-like" patterns in the discussion, which suggests a reaction-diffusion system (e.g. diffusible activators and inhibitors that act on cells and regulate their own secretion) to spontaneously form patterns. This is misleading in at least two ways:

-The authors spend most of the manuscript arguing that the pattern emerges through a cell sorting/cell migration mechanism, entirely different than patterning via reaction-diffusion on a fixed field of cells.

-The experimental system does not involve stochastic symmetry breaking in the classic sense of what "Turing-like" patterns purport to explain. In a Turing pattern, small fluctuations in an otherwise uniform field of cells are amplified by feedback to form bifurcated states and spatial patterns. Here, the inhomogeneity is "baked in" directly by the 1:1 co-culture and light stimulation.

-Perhaps very dim or transient illumination, leading to beta-catenin levels that are only very slightly above wild-type, would also self-amplify into distinct domains. This scenario would be more plausibly explained by Turing-like feedback to amplify small asymmetries into large spatial domains. The authors do not need to carry out this experiment, but it would lend weight to a Turing-like model if that is a claim they feel strongly about making.

Based on these arguments, I feel that it would be worth pointing out in the Discussion that these results are not "Turing-like" in themselves (either in mechanism or conceptual framework). At the very least, it should clearly consider these points and refute them, rather than make the unsupported claim.

1. Thank you for this insightful comment. We agree with this reasoning and have revised the Discussion section text to clarify this point and address your concern, reproduced below:

"With the hESC co-culture approach, we demonstrated that in the absence of directional cues, such as gradients of Wnt signaling and morphogen secretion from extraembryonic tissues, heterogeneous activation of Wnt is sufficient for self-organization and mesendoderm migration away from an epithelial cell population. Unlike classic Turing systems, cell-to-cell variability in WT/optoWnt co-cultures does not self-emerge but rather is directly controlled with optogenetic stimulation and leads to tissue-scale cell segregation and patterning. Consequently, emergence of cell-to-cell heterogeneity in Wnt signaling may contribute to tissue organization."

(2) I am not fully convinced by the authors' claim that Nodal/TGFbeta signaling is essential for symmetry breaking. I don't want to suggest that the authors have to answer every question below, but at least some picture of what is going on (and how Nodal is involved) would be helpful:

Thank you for these comments. To address the role of Nodal/TGF-□ signaling in cell segregation, we repeated inhibitor treatments with Nodal-TGF-□ receptor inhibitor SB431542, as well as with BMP receptor inhibitors and Wnt secretion inhibitors, and analyzed the effect on cell segregation, differentiation, and EMT. The results of these experiments are included as an additional main figure (Figure 5) and supplementary figure S5B.

-Figure 4I is the strongest data that Nodal signaling is important, but is hard to interpret. Although the clumps of opto-Wnt cells are smaller, sorting into distinct domains is still evident!

We repeated inhibitor treatments with Nodal-TGF-□ receptor inhibitor SB431542 and added additional quantification and images (Figure 5B-D, Figure S5B). While BMP and Wnt secretion inhibition showed no significant abrogation of self-organization, Nodal-TGF-□ receptor inhibition showed a striking decrease in cell segregation, where optoWnt aggregates were barely detectable and had a significantly decreased abundance and size. As the reviewer notes, there appeared to

© 2023. Published by The Company of Biologists under the terms of the Creative Commons Attribution License (https://creativecommons.org/licenses/by/4.0/).

be a small amount of residual optoWnt clustering,
however SB treatment resulted in no distinct boundaries between WT and optoWnt domains, no detectable optoWnt cell piling into vertical stacks, and no apparent contact area minimization between domains. Additional Supplementary images are included to visualize changes in aggregation morphology (S5B).

-How is Nodal's effect supposedly mediated? Does sorting initiate similarly but arrest earlier in Nodal-inhibited cells? Does Nodal inhibition affect opto-Wnt cells, wild-type cells, or both? Could the results of 4J-K simply be explained by some baseline cell death due to nodal inhibition decreasing proliferation, or a change in overall cell motility but not the sorting program?
-Do Nodal-inhibited OptoWnt cells still show changes in Ecad/Snail expression? (If so, this would suggest a mechanism for its action. If not, it is very hard to understand what role it might play to specifically regulate cell sorting.)

We hypothesized that initial segregation is mediated by the strong cell state changes of activated optoWnt cells undergoing differentiation and EMT/migration. To elucidate how Nodal's effect is mediated, we quantified downstream effects of inhibition on pSMAD phosphorylation, differentiation via Bra immunostaining, and EMT via ECAD and NCAD immunostaining. We observed no detectable pSMAD1/5 or pSMAD2/3 expression in unilluminated cultures, indicating that induction occurred due to feedback downstream of optoWnt stimulation without baseline induction from culture media. As expected, BMP inhibitor treatment specifically reduced pSMAD 1/5 to unilluminated levels, whereas Nodal- TGF-¤ inhibitor treatment specifically reduced pSMAD 2/3. Thus, though illumination stimulated both SMAD1/5 and SMAD2/3 phosphorylation in optoWnt cells, only inhibition of pSMAD2/3 signaling resulted in a decreased cell segregation phenotype.

Interestingly, optoWnt cells were able to acquire Bra expression under SB treatment, with no difference in Bra nuclear intensity compared with untreated co-cultures (Figure 5H, 5I). SB treatment also did not show an observable effect on cell proliferation, as measured with Ki67 staining (Supplementary Figure S11G). Furthermore, optoWnt cells were also able to undergo the cadherin expression switch from ECAD to NCAD (Figure 5G, 5H), suggesting that SB treatment did not prevent initial stages of EMT. However, though expression of

ECAD decreased under SB treatment, there was still detectable expression suggesting an incomplete EMT (Figure 5J). Indeed, changing the timing of SB treatment to the final 24 hrs of light stimulation (24 – 48 hrs) similarly abrogated segregation (Figure 5K-L), suggesting that segregation can be inhibited even when cells have initiated differentiation and EMT. Instead, this 24 – 48 hrs period corresponds to the time window wherein optoWnt cells become most migratory (Figure 4E-F), suggesting that TGF-¤ signaling feedback is necessary to maintain migration of optoWnt cells and to induce cell segregation. These results suggest that TGF-¤ signaling feedback is necessary for optoWnt cells to fully undergo EMT and to induce migration of optoWnt cells. In conclusion, segregation of co- cultures required both optoWnt activation and downstream Nodal-TGF-¤ feedback, which may play a role in maintaining optoWnt cell migration.

-Is Smad phosphorylation equally induced in both wild-type and optoWnt cells to equal extents during co-culture? The authors only quantify the effect in wild-type cells, not optoWnt cells, in Figure 5F-G. However, the inset images indicate uniform Smad activation in both cell populations, suggesting that Nodal signaling is uniformly induced in all cells. The authors should quantify Nodal in the two cell populations during co-culture as in Figure 5F-G.
-Figure 5G may show a statistically significant effect, but its magnitude is so small (~0.18 AU vs ~0.2 AU) that it is hard to interpret as biologically meaningful.

Further, to address the question of whether SMAD phosphorylation is equally induced in both WT and optoWnt cells, we quantified pSMAD1/5 and pSMAD2/3 in both WT and optoWnt cells under control as well as TGF-¤ and BMP inhibitor treatment (please see the new Figures 6F, 6G, 6H, 6I). We found that optoWnt activation induces pSMAD1/5 and pSMAD2/3 in both WT and optoWnt cells, with slightly higher induction in optoWnt cells. treatment with BMP inhibitors (LDN and Noggin) and Nodal-TGF-ß (SB) similarly showed respective reduction of pSMAD1/5 and pSMAD2/3 levels in WT cells. Such signal inhibition at the receptor level supports the hypothesis that WT cells display activated BMP/TGF-¤ signaling as a result of signaling from optoWnt cells in co-culture.

© 2023. Published by The Company of Biologists under the terms of the Creative Commons Attribution License (https://creativecommons.org/licenses/by/4.0/).

Figure 5

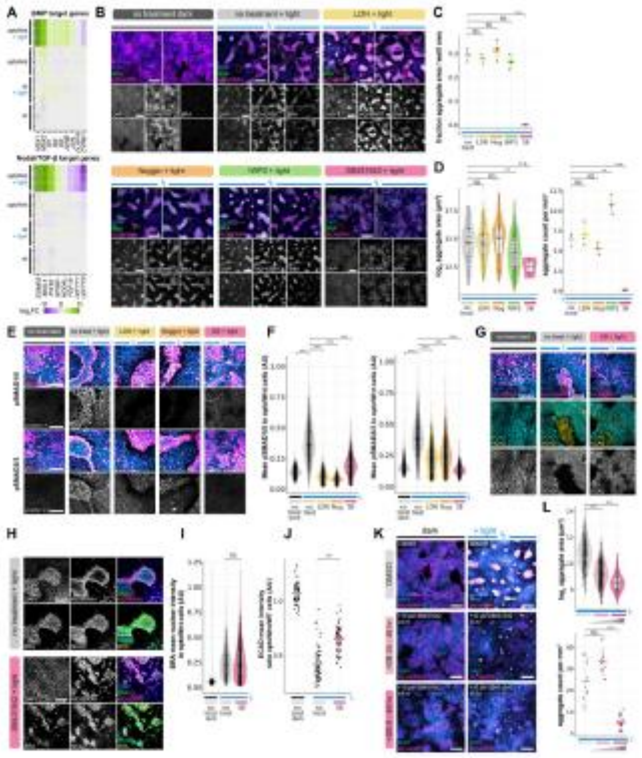

Supplementary Figure 5

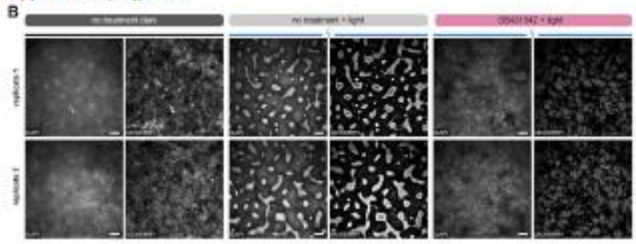

Figure 6

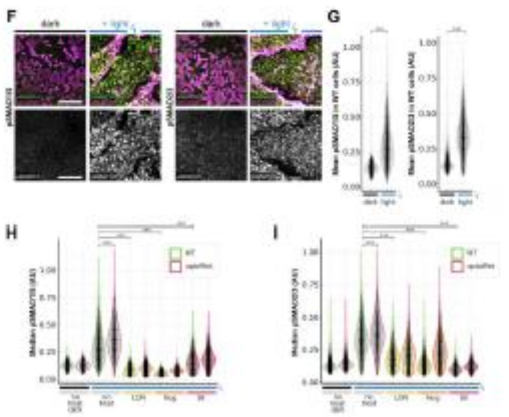

(3) The authors claim that their data suggest "that Wnt alone is sufficient for initiating the human BMP4-Wnt-Nodal primitive streak feedback loop without the need for exogenous BMP4 stimulation". I cannot say for sure whether this has ever been reported in hESCs, but certainly

© 2023. Published by The Company of Biologists under the terms of the Creative Commons Attribution License (https://creativecommons.org/licenses/by/4.0/).

this well established in mouse ESCs, for instance in Martinez-Arias' early gastruloid papers, which robustly develop A-P axes in response to CHIR treatment without BMP but are not cited in the Discussion section related to this point.

3. Thank you for this comment. Indeed, Wnt signaling is sufficient for mouse ESC axis patterning as evidenced by mouse gastruloid and 2D mouse micropattern systems. However, in human, the contribution of Wnt / BMP / Nodal signaling is less well understood. Self-organizing human systems typically use BMP4 signal modulation, where it has been shown that Wnt activation occurs downstream of BMP4, or alternatively a combined Wnt and Activin treatment to induce A-P axis patterning, particularly in micropattern cultures e.g. *Warmflash et al Nature Methods 2014, Etoc et al Developmental Cell 2016, Martyn et al Nature 2018, Simunovic et al Nature Cell Biology 2019, Chhabra et al Plos Biol 2019*. The more recent development of a 3D human gastruloid system (*Moris et al Nature 2020*) implements a pre-treatment of hESCs with CHIR prior to aggregate formation, suggesting that cell priming with Wnt signal activation is necessary for axis patterning. Interestingly, however, neither Wnt3a or BMP4 treatment alone was sufficient for gastruloid formation and could not substitute CHIR treatment, suggesting that molecular effects of CHIR differ from those of canonical Wnt ligands. It has thus remained unclear whether Wnt stimulation alone is sufficient for inducing self-organized axis patterning and downstream BMP / Nodal feedback in human cultures. We updated the Discussion text to help clarify this point and added the Martinez Arias reference, as reproduced below:

"Indeed, optogenetic stimulation of Wnt resulted in EMT signatures consistent with the mouse epiblast (Martyn et al., 2018; Sumi et al., 2008) and downstream activation of both BMP and Nodal/TGF-□ signaling following 48 hrs of illumination (Figure 5E-F). This suggests that Wnt alone is sufficient for initiating the human BMP4-Wnt-Nodal primitive streak feedback loop without the need for exogenous BMP4 or Activin stimulation (Ben-Haim et al., 2006; Chhabra et al., 2019; Etoc et al., 2016; Martyn et al., 2018; Simunovic et al., 2019; Warmflash et al., 2014; Xu et al., 2021), as observed in mouse in vitro models (Turner et al., 2017)."

(4) The authors discuss EMT at length, but this might invite considerable controversy from the ES cell field, as many authors are careful to avoid suggesting a true "epithelial-to- mesenchymal" transition because embryonic stem cells do not form a true epithelium, and instead refer to an EsMT (embryonic stem cell to mesenchymal transition); see e.g. Cermola et al, Stem Cell Rep 2022. It might be prudent to amend the terminology used for this transition.

4. Thank you for this comment. We find the EMT terminology to be commonly used in the gastruloid and synthetic embryoid fields to describe the mesenchymal transition of embryonic stem cells during mesendoderm differentiation and axis patterning. It is used for example in *Simunovic et al Nature Cell Biology 2019, Martyn et al Nature 2018, Girgin et al Nature Communications 2021, ten Berge et al Cell Stem Cell 2008, Harrison et al Science 2017, Sozen et al Nature Cell Biology 2018, Amadei et al Nature 2022*. Though we agree that there are molecular differences between the EMT of *in vitro* synthetic systems and *in vivo* embryonic epiblast, we hesitate to amend the terminology and prefer to use the more commonly used term of EMT.

Second decision letter

MS ID#: DEVELOP/2022/201386

MS TITLE: Optogenetic control of Wnt signaling models cell-intrinsic embryogenic patterning using 2D human pluripotent stem cell culture

AUTHORS: Nicole A Repina, Hunter J Johnson, Xiaoping Bao, Joshua A Zimmermann, David A Joy, Shirley Z Bi, Ravi S Kane, and David V Schaffer

© 2023. Published by The Company of Biologists under the terms of the Creative Commons Attribution License (https://creativecommons.org/licenses/by/4.0/).

I have now received all the referees reports on the above manuscript, and have reached a decision. The referees' comments are appended below, or you can access them online: please go to BenchPress and click on the 'Manuscripts with Decisions' queue in the Author Area.

The overall evaluation is positive and we would like to publish a revised manuscript in Development, provided that the referees' comments can be satisfactorily addressed. Please attend to all of the reviewers' comments in your revised manuscript and detail them in your point-by-point response. If you do not agree with any of their criticisms or suggestions explain clearly why this is so. If it would be helpful, you are welcome to contact us to discuss your revision in greater detail. Please send us a point-by-point response indicating your plans for addressing the referee's comments, and we will look over this and provide further guidance.

Reviewer 1

Advance summary and potential significance to field

In this manuscript, the authors use an optogenetic system to control the activation of Wnt signaling intracellular cascade (optoWnt). They use this system to study how the cell-intrinsic variability of Wnt activity in a human Pluripotent Stem Cells (hPSC) population contributes to self-organization reminiscent of primitive streak behavior during gastrulation.

The authors show that:
1) Activation of Wnt signaling pushes hPSC towards a primitive streak (PS)/mesendodermal cell fate and EMT as shown in many publications.
2) Upon activation of Wnt pathway cell sorting occurs: Wnt-induced cells set apart from the wild-type cells (non Wnt-induced).
3) The sorting of Wnt-active PS cells seems to be due to the activation of an EMT program and to increased cell motility. The authors show that the cell sorting mechanism is dependent on TGFb activity downstream of Wnt pathway activation.
Of note, differential cell proliferation does not seem to play a role in cell sorting.
4) It seems that the secretion of BMP ligands from the Wnt-induced cells triggers a BMP response in the wild-type (non Wnt-induced) cells. The authors suggest that BMP signals from the PS cells could contribute to a cell priming effect of epiblast cells for subsequent cell specification.

In my opinion, the manuscript is well written and the experiments are very well controlled. OptoWnt is a useful tool for dissecting intercellular mechanisms that contribute to human early development. In summary, I find that the manuscript fits the interest of this scientific journal.

Comments for the author

I want to congratulate the authors for the experiments they performed to answer all my points. I do not have any further questions.
I just have one small comment on point 7 reviewer 1: also in the paper “Warmflash A. et al, Nat. Methods, 2014, DOI: 10.1038/nmeth.3016” a hPSC maintenance medium (mTeSR1) has been used during the treatment of hESC with BMP ligands. In that case primitive streak differentiation has been observed. This is in contrast with the idea proposed by the authors that the presence of TGFb and FGF2 could overpower the differentiation effect of BMP on the WT cells.
In my opinion, the manuscript in its current status is worth of publication on this journal.
My best regards,
Andrea Manfrin.

Reviewer 2

Advance summary and potential significance to field

This is a revision of an already-excellent manuscript, and I congratulate the authors on having addressed all of my prior concerns.

© 2023. Published by The Company of Biologists under the terms of the Creative Commons Attribution License (https://creativecommons.org/licenses/by/4.0/).

(summary from prior revision): In this study, Repina and coworkers study the consequences of light-induced Wnt signaling in human embryonic stem cells (hESCs). Both the optoWnt optogenetic tool and the engineered optoWnt hESC line used in this study were previously published, but prior studies have not used these optogenetics tools to examine the question of how cells with different levels of Wnt activity might self-organize into distinct domains. Broadly, the authors find that a heterogeneous mixture of optoWnt and WT cells segregates into distinct domains, presumably through an EMT-like process and cell sorting by differential adhesion. I have been aware of this interesting work for some time since it became available as a preprint and am very happy to see it considered for publication at Development. The paper is well-written, the data solid, and the findings of broad interest to a developmental audience.

Comments for the author

I have no further experimental revisions to suggest. However, I have one minor suggestion involving a new piece of data added during revision where I am not sure I understand / agree with the authors' interpretation:
In Figure 4G-I, the authors describe the result when a mixture of OptoWnt/WT cells are treated with ROCK inhibitor. They describe the result by saying the cultures "did not display the apparent contact area minimization between the optoWnt and WT domains (Figure 4I)."
My confusion is that ES cell cultures of this type are not strictly two-dimensional. In a strictly two-dimensional system, it is certainly the case that the smaller, circular domains formed in the ROCKi treatment condition would have a greater OptoWnt colony perimeter to colony area, increasing the contact surface for a fixed number of OptoWnt cells. But ES cells often grow into 3D spheroids even in adherent culture, and I suspect that the "circular" colonies the authors observe are hemispheres that can reduce interfacial area by growing in the z-direction. This hypothesis would be consistent with the authors' results in Fig 4H, in which ROCKi treatment leads to lesser overall surface area in the 2D plane.
I would argue that the authors should either quantify the actual contact area between OptoWnt and WT domains from 3D z-stack images in the untreated vs ROCKi treatment cases or modify their interpretation of this one experiment in the text.

Second revision

Author response to reviewers' comments

Reviewer 1

I want to congratulate the authors for the experiments they performed to answer all my points. I do not have any further questions.

I just have one small comment on point 7 reviewer 1: also in the paper "Warmflash A. et al, Nat. Methods, 2014, DOI: 10.1038/nmeth.3016" a hPSC maintenance medium (mTeSR1) has been used during the treatment of hESC with BMP ligands. In that case primitive streak differentiation has been observed. This is in contrast with the idea proposed by the authors that the presence of TGFb and FGF2 could overpower the differentiation effect of BMP on the WT cells.

Thank you for this comment. Indeed, the reported mesendoderm differentiation in the referenced Warmflash et al. paper is achieved using high doses of BMP-4 (50 ng/mL), and likely the cell fate response is dependent on the concentration of BMP in relation to the mTeSR1 media factors (e.g. FGF2 and TGF-?). Of note, the cellular state of the starting cultures is also likely different from our optoWnt/WT setup due to differing pre-culture conditions, plate coating, and lack of micropattern geometry.

© 2023. Published by The Company of Biologists under the terms of the Creative Commons Attribution License (https://creativecommons.org/licenses/by/4.0/).

Reviewer 2

I have no further experimental revisions to suggest. However, I have one minor suggestion involving a new piece of data added during revision where I am not sure I understand / agree with the authors' interpretation:

In Figure 4G-I, the authors describe the result when a mixture of OptoWnt/WT cells are treated with ROCK inhibitor. They describe the result by saying the cultures "did not display the apparent contact area minimization between the optoWnt and WT domains (Figure 4I)."

My confusion is that ES cell cultures of this type are not strictly two-dimensional. In a strictly two-dimensional system, it is certainly the case that the smaller, circular domains formed in the ROCKi treatment condition would have a greater OptoWnt colony perimeter to colony area, increasing the contact surface for a fixed number of OptoWnt cells. But ES cells often grow into 3D spheroids even in adherent culture, and I suspect that the "circular" colonies the authors observe are hemispheres that can reduce interfacial area by growing in the z-direction. This hypothesis would be consistent with the authors' results in Fig 4H, in which ROCKi treatment leads to lesser overall surface area in the 2D plane.

I would argue that the authors should either quantify the actual contact area between OptoWnt and WT domains from 3D z-stack images in the untreated vs ROCKi treatment cases or modify their interpretation of this one experiment in the text.

We thank the reviewer for this point, and we have amended the manuscript text as shown below. Untreated cultures show a spatial separation between WT and optoWnt domains (Fig. 3C, 4G, with a clear gap between cells at the domain boundary), whereas we noted that under ROCKi treatment this separation is reduced, so that WT cells are in closer contact with OptoWnt cells at the domain boundary. We had described this spatial separation as "contact area minimization", though we understand that this term is misleading and have now rephrased the text.
Indeed, both in untreated and ROCKi-treated cultures, the activated optoWnt domains grow in the z-direction, as the reviewer remarks.

"That is, though optoWnt and WT cells still segregated, treatment with Y-27632 resulted in smaller, more abundant, and more circular optoWnt aggregates that displayed a less distinct spatial separation at the boundary edges between the optoWnt and WT domains."

Third decision letter

MS ID#: DEVELOP/2022/201386

MS TITLE: Optogenetic control of Wnt signaling models cell-intrinsic embryogenic patterning using 2D human pluripotent stem cell culture

AUTHORS: Nicole A Repina, Hunter J Johnson, Xiaoping Bao, Joshua A Zimmermann, David A Joy, Shirley Z Bi, Ravi S Kane, and David V Schaffer
ARTICLE TYPE: Research Article

I am happy to tell you that your manuscript has been accepted for publication in Development, pending our standard ethics checks.

Reviewer 1

Advance summary and potential significance to field

In this manuscript, the authors use an optogenetic system to control the activation of Wnt signaling intracellular cascade (optoWnt). They use this system to study how the cell-intrinsic variability of

© 2023. Published by The Company of Biologists under the terms of the Creative Commons Attribution License (https://creativecommons.org/licenses/by/4.0/).

Wnt activity in a human Pluripotent Stem Cells (hPSC) population contributes to self-organization reminiscent of primitive streak behavior during gastrulation.
The authors showed that:

1)Activation of Wnt signaling pushes hPSC towards a primitive streak (PS)/mesendodermal cell fate and EMT as shown in many publications.
2) Upon activation of Wnt pathway cell sorting occurs: Wnt-induced cells set apart from the wild-type cells (non Wnt-induced).
3) The sorting of Wnt-active PS cells seems to be due to the activation of an EMT program and to increased cell motility. The authors show that the cell sorting mechanism is dependent on TGFb activity downstream of Wnt pathway activation.
Of note, differential cell proliferation does not seem to play a role in cell sorting.
4) It seems that the secretion of BMP ligands from the Wnt-induced cells triggers a BMP response in the wild-type (non Wnt-induced) cells. The authors suggest that BMP signals from the PS cells could contribute to a cell priming effect of epiblast cells for subsequent cell specification.

In my opinion, the manuscript is well written and the experiments are well controlled. OptoWnt is a useful tool for dissecting intercellular mechanisms that contribute to human early development. In summary, I find that the manuscript fits the interest of this scientific journal and it is definitely worth of consideration.

Comments for the author

I want to thank the authors for their reply.
I have no further questions nor comments.
The manuscript is in my opinion worth of publication in Development.
Andrea Manfrin.

© 2023. Published by The Company of Biologists under the terms of the Creative Commons Attribution License (https://creativecommons.org/licenses/by/4.0/).
